# Refinement of X-ray and electron diffraction crystal structures using analytical Fourier transforms of Slater-type atomic wavefunctions in *Olex2*

**DOI:** 10.1107/S1600576723010981

**Published:** 2024-02-01

**Authors:** Florian Kleemiss, Norbert Peyerimhoff, Michael Bodensteiner

**Affiliations:** a Institut für Anorganische Chemie, RWTH Aachen University, Landoltweg 1a, 52074 Aachen, Germany; b Universität Regensburg, Universitätsstraße 31, 93053 Regensburg, Germany; c University of Durham, Stockton Road, Durham DH1 3LE, United Kingdom; Ecole National Supérieure des Mines, Saint-Etienne, France

**Keywords:** refinement, electron diffraction, X-ray diffraction

## Abstract

An implementation of Slater-type spherical scattering factors for X-ray and electron diffraction for elements in the range *Z* = 1–103 is presented within the software *Olex2*. Both high- and low-angle Fourier behaviour of atomic electron density and electrostatic potential can thus be addressed, in contrast to the limited flexibility of the four Gaussian plus constant descriptions which are currently the most widely used method for calculating atomic scattering factors during refinement. The implementation presented here accommodates the increasing complexity of the electronic structure of heavier elements by using complete atomic wavefunctions without any interpolation between precalculated tables or intermediate fitting functions. Atomic wavefunctions for singly charged ions are implemented and made accessible, and these show drastic changes in electron diffraction scattering factors compared with the neutral atom. A comparison between the two different spherical models of neutral atoms is presented as an example for four different kinds of X-ray and two electron diffraction structures, and comparisons of refinement results using the existing diffraction data are discussed. A systematic but slight improvement in *R* values and residual densities can be observed when using the new scattering factors, and this is discussed relative to effects on the atomic displacement parameters and atomic positions, which are prominent near the heavier elements in a structure.

## Abstract 2

Analytical scattering factors for X-ray and electron diffraction from spherical Slater-type orbitals are implemented in *Olex2* to overcome shortcomings of interpolation and fitting methods and compare refinement results.

## 1. Introduction

Advances in non-spherical refinement techniques to describe the electron density of a model in crystallographic least-squares refinement have allowed detailed analysis of various types of structures (Kleemiss *et al.*, 2021 ▸), even when the data quality is not sufficient for classical charge-density fitting, *e.g.* by multipole refinement (Coppens *et al.*, 1979 ▸; Hansen & Coppens, 1978 ▸). There is no reasonable argument for using spherical atomic electron densities other than speed and low computational cost. Sometimes, a lack of infrastructure for handling non-spherical models makes it desirable to revert to using spherical atomic form factors in the refinement. Such a treatment can be done for isolated parts of a crystallographic model, for example for disordered regions or solvents, to save time and computational resources within the framework of combined models, as recently introduced within *Olex2* (Jha *et al.*, 2023 ▸) or in multipole models upon selection of solely monopole populations for individual atoms. The description of atoms in standard refinement engines used for building spherical atom models is, following the recommendations of *International Tables for Crystallography* (Maslen *et al.*, 2006 ▸), a sum of Gaussian functions fitted to precalculated scattering factors as given by Cromer & Mann (1968 ▸) and Cromer & Waber (1965 ▸), themselves calculated for example by Cromer & Mann (1967 ▸) on the basis of Hartree–Fock wavefunctions calculated by Mann (1967 ▸, 1968 ▸). The Gaussian functions are often extended by adding a constant. These can be found in, and are used automatically by, software such as *olex2.refine* (Dolomanov *et al.*, 2009 ▸) or *SHELXL* (Sheldrick, 2015 ▸)

**Figure 1:**
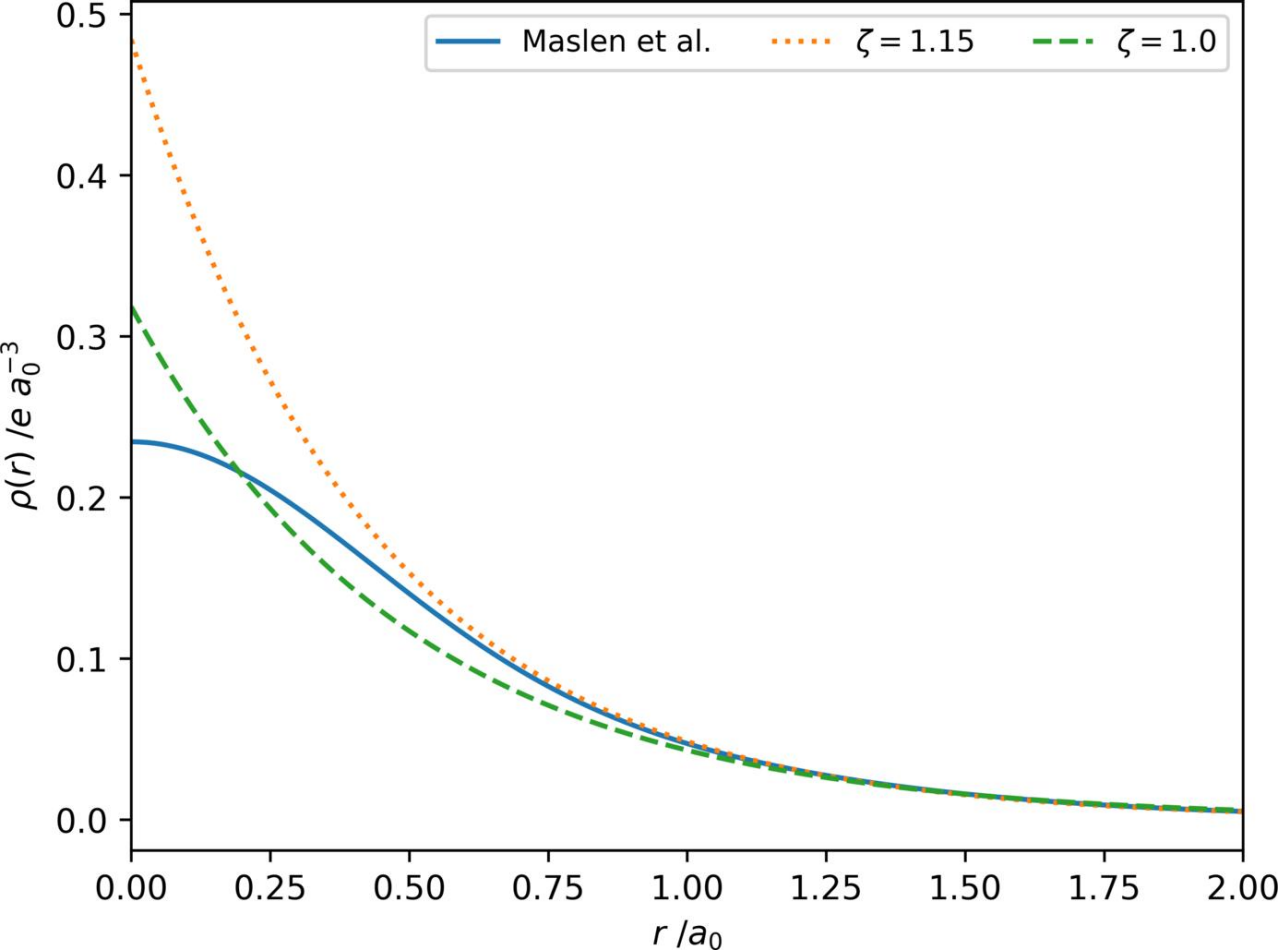
A plot of the electron density using different descriptions at a distance *r* in atomic units from a hydrogen atom. The solid blue line represents the electron density according to the Gaussian functions of Maslen *et al.* (2006 ▸), the orange dotted line represents the electron density when employing a single Slater-type function with an exponent of 1.15 in agreement with the scattering factor of Stewart *et al.* (1965 ▸) and the green dashed line shows the electron density of a hydrogen atom in the unbound state using a Slater-type function.

While these functions prove to be very useful for fast determinations of atomic connectivity, there are downsides, and a need for re-evaluation has recently been shown in the literature (Thorkildsen, 2023 ▸; Olukayode *et al.*, 2023*a* ▸,*b* ▸), This requirement has led to a variety of developments in recent years, ranging from high-quality X-ray scattering factors from freshly calculated wavefunctions to relativistically corrected electron diffraction scattering factors (Thorkildsen, 2023 ▸; Olukayode *et al.*, 2023*a* ▸,*b* ▸; Yonekura *et al.*, 2018 ▸; Lentzen, 2019 ▸). The main reason for the routine use of Gaussian-type functions in crystallography is the same as in quantum mechanical computations: the required calculations are more straightforward to perform and require less computational power, making them attractive even though a trade-off in accuracy must be made (Magalhães, 2014 ▸). However, no number of contracted Gaussian functions will give the correct behaviour at small distances from the atomic position known from quantum mechanics: a cusp of the radial function at the nucleus. Fig. 1 ▸ shows this difference between the electron densities of a hydrogen atom. For a more detailed discussion of the issue, we refer the reader to Magalhães (2014 ▸).

The three-dimensional Fourier transform of a Gaussian function is itself a Gaussian; the constant of the classical atomic scattering factors becomes a delta distribution. The Fourier transform of an electron density calculated from Slater-type orbital functions with higher exponents of the radius *r* is a rational function with polynomials of increasing degree in the numerator and denominator as the exponent of the distance of the radial electron-density function increases [see equation (1) and the supporting information]. In particular, the long-range behaviour of Slater functions makes a difference in the calculated intensities of low-order reflections during crystallographic refinement due to the reciprocal relationship between the distance from the atomic position and the length of the scattering vector. The cusp at the other end of the nuclear position introduces a difference in calculated intensities for higher-order reflections, especially compared with the constant term of the Gaussian fit. The effect of the cusp can be seen in the deviation between relativistic Slater-type wavefunctions and a fit of a series of Gaussian functions being used to calculate scattering factors, as done, for example, by Macchi & Coppens (2001 ▸), where the difference in scattering factors of non-relativistic wavefunctions systematically increases with sin(θ)/λ.

**Figure 2:**
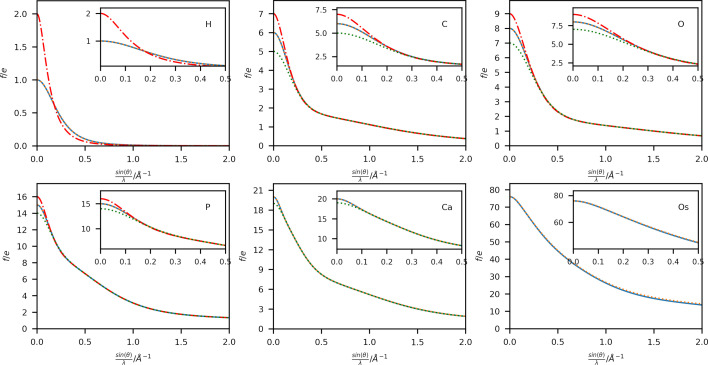
Plots of X-ray scattering factors in electrons obtained by 4G+c (orange dashed lines; Maslen *et al.*, 2006 ▸) and Thakkar wavefunctions (neutral: solid blue lines, cation: green dotted lines, anion: red dashed–dotted lines) for elements (top left to bottom right) H, C, O, P, Ca and Os against sin(θ)/λ in Å ^−1^.

**Figure 3:**
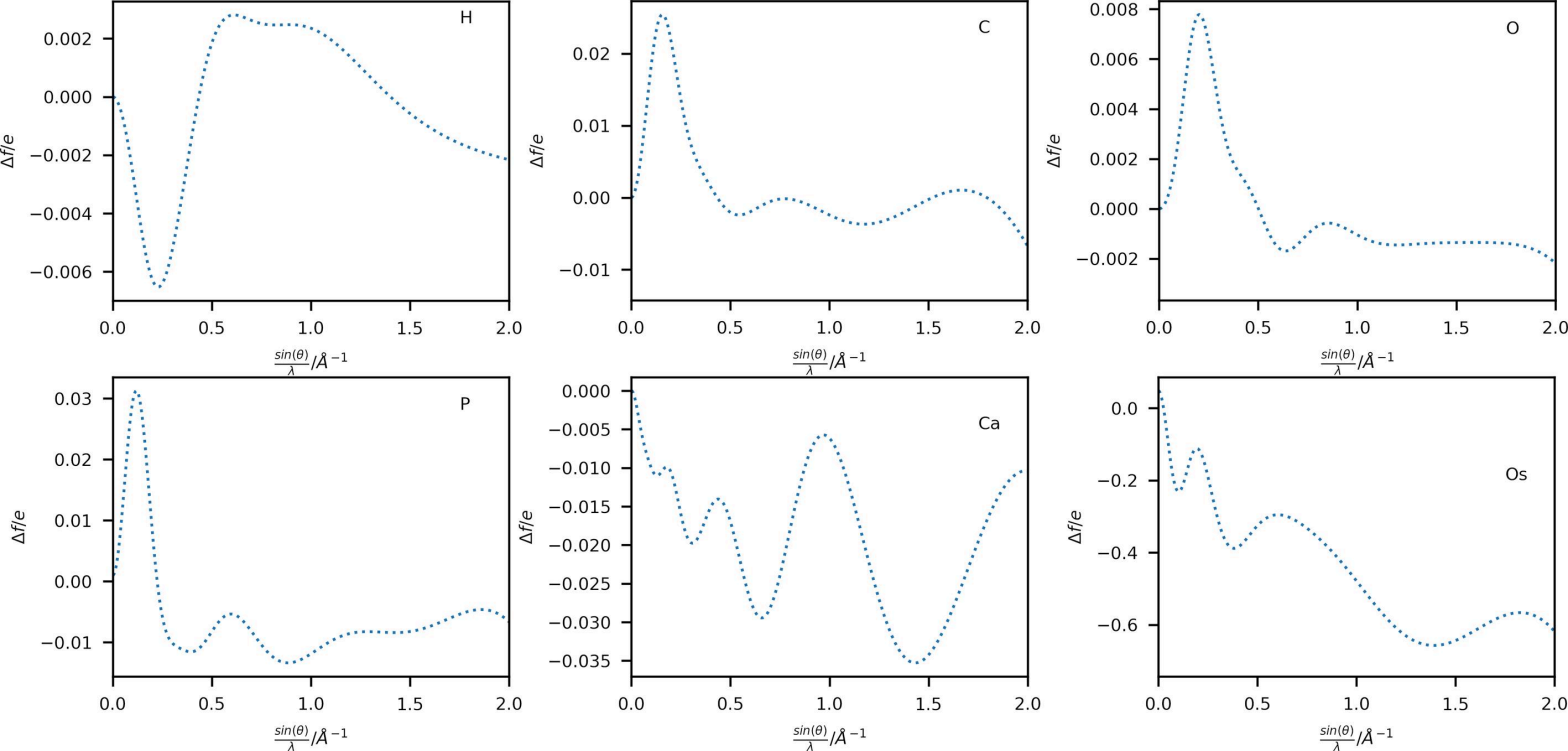
Difference plots between Thakkar-based X-ray scattering factors and those obtained by 4G+c (Maslen *et al.*, 2006 ▸) in electrons for neutral atoms of elements (top left to bottom right) H, C, O, P, Ca and Os against sin(θ)/λ in Å ^−1^.

Routinely available high-quality data from laboratory experiments on modern diffractometers show growing discrepancies between models and data, *i.e.* refinement statistics such as *R* values and structured residual densities that cannot be disregarded as noise. In particular, elements with *Z* > 35 exhibit large residual electron-density values after refinement using both spherical and non-spherical electron-density models. Examples exhibiting the structured appearance of these residual electron-density distributions, even after non-spherical refinements, were presented in recent studies for Hg, Os or Au (Malaspina *et al.*, 2019 ▸; Kleemiss *et al.*, 2021 ▸; Pawlędzio *et al.*, 2021 ▸, 2022 ▸). The increasing threshold for residual electron-density values of structures containing heavier elements before a warning is triggered during data validation procedures by the IUCr *CheckCIF* procedure (https://checkcif.iucr.org/) tries to address these growing discrepancies during structure validation. For example, the tests DIFMX01 and DIFMN02 scale with the highest atomic number in the model. This assumption seems plausible since a higher absolute value of densities leads to higher absolute values of the random noise. However, the systematic concentric distribution of the residual electron density around the heaviest scatterer is remarkably similar to the distribution of the electron-density differences between Gaussian- and Slater-based models. Refinements with smaller residuals around heavy elements require a model using more accurate scattering factors that capture both high- and low-angle Fourier behaviour, presented here. The precision is improved compared with the limited flexibility of the four Gaussian plus constant descriptions (4G+c), currently the most widely used method for calculating atomic scattering factors (Figs. 2 and 3, Os example; Maslen *et al.*, 2006 ▸). The implementation presented in this work accommodates the increasing complexity of the electronic structure of heavier elements by using complete atomic wavefunctions without any interpolation between precalculated tables or intermediate fitting functions.

There are several different sets of scattering factors from Slater functions available, *e.g.* based on the atomic functions of Clementi & Roetti (1974 ▸), McLean & McLean (1981 ▸), Macchi & Coppens (2001 ▸) or most recently Olukayode *et al.* (2023*a* ▸,*b* ▸). The wavefunctions underlying these scattering factors differ regarding the relativistic corrections and the size of the basis sets used in the calculations. The mentioned scattering factors are available in different software, either in specially written code for the calculation, as in the case of Olukayode and co-workers, or in multipole refinement software like *XD2016* (Volkov *et al.*, 2016 ▸) or *MoPro* (Guillot *et al.*, 2001 ▸). However, there is no interface for Slater-type scattering factors in software such as *Olex2* or *cctbx*, which are well established in modelling with spherical scattering factors (Dolomanov *et al.*, 2009 ▸; Bourhis *et al.*, 2015 ▸; Grosse-Kunstleve *et al.*, 2002 ▸).

## 2. Calculation of scattering factors from Slater-type atomic wavefunctions

Coefficients of tabulated Slater-type orbital wavefunctions from Thakkar, Koga and co-workers are conveniently available over the periodic table for *Z* = 1–103 and provide accurate descriptions of the atomic electron density (Koga *et al.*, 1999 ▸, 2000 ▸). The availability of the ionic wavefunctions of singly charged cations and anions from the same work is shown in Table S1. These Hartree–Fock Slater-type wavefunctions were generated using a building scheme based on two criteria: angular momentum *l* and the number of occupied orbitals with that angular momentum 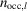 . For this atom specific pair 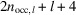 radial Slater-type functions are used, with a few exceptions as mentioned in the original publications for elements to *Z* = 54; elements *Z* = 55–103 use 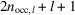 corresponding functions (Koga *et al.*, 1999 ▸, 2000 ▸). The choice of minimization function in their work was based on constraints to represent the nuclear cusp, long-range behaviour and virial ratio (Koga *et al.*, 1999 ▸). The electron density of an atom at a distance *r* can be calculated using the wavefunction coefficients from the reported wavefunctions according to

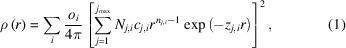

where *i* runs over all the orbitals (1*s*, 2*s*, 3*s*,…, 7*s*, 2*p*, 3*p*,…, 7*p*, 3*d*,…, 6*d*, 4*f*, 5*f*), *j* denotes the number of coefficients of a Slater-type primitive for this contracted orbital with 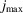 coefficients and *o_i_* denotes the occupation of said orbital, *N*_*j*,*i*_ is the normalization constant, *c*_*j*,*i*_ is the tabulated atomic orbital coefficient, *n*_*j*,*i*_ is the order of the radial function, and *z*_*j*,*i*_ is the exponent. The calculation of an atomic scattering factor can easily be obtained by applying a three-dimensional Fourier transform of this function. The resulting expression can be solved analytically (see the supporting information for details), as in the case of the multipole formalism for the monopole contribution to scattering (Avery & Watson, 1977 ▸; Maslen *et al.*, 2006 ▸):

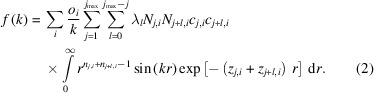

Here, 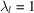 for *l* = 0 and 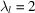 for *l* ≥ 1, where *l* is a second iterator over the exponents of a contracted orbital and *k* is the length of the scattering vector. The integral can be evaluated analytically by a recursion of the appearing sin and cos integrals using partial integration:

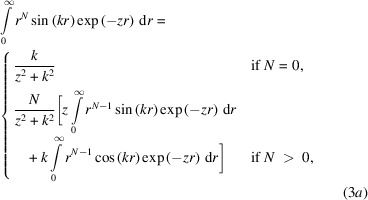




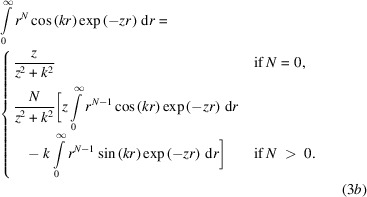




With these relations, it is straightforward to calculate the spherical scattering factor from the tabulated wavefunction without compromising using a series of fitted Gaussian functions or interpolations from tabulated values. Care must be taken when using the exponents of the tabulated wavefunctions to convert them to the units used for the lengths of the scattering vector *k*, since many wavefunctions are reported in units of bohr while scattering vectors are usually given in units of ångströms. The resulting expressions match the functions reported for application in the multipole model in Table 6.1.1.9 of *International Tables* (Maslen *et al.*, 2006 ▸).

In addition, the availability of analytical scattering factors for all elements and many of their ions allows for the calculation of scattering factors at the same level of theory for use in electron diffraction (ED) experiments. A comparison between the scattering factors calculated by Peng (1999 ▸) and those obtained by the Mott–Bethe transformation (Bethe, 1930 ▸; Mott, 1930 ▸) of the newly presented Thakkar-based scattering factors, following the procedure described in equation (11) of Peng (1999 ▸), is presented here and comparisons of refinement results of existing diffraction data are discussed. The relationship between the X-ray and electron scattering factors is reported to be

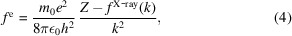

where *f* ^e^ is the electron diffraction scattering factor, *f* ^X-ray^ is the scattering factor obtained by equation (2) and *Z* is the atomic number for which the scattering factor is calculated; *m*_0_, *e*, 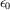 and *h* refer to the natural constant of the electron mass, the electron charge, the permittivity of a vacuum and the Planck constant, respectively.

## 3. Implementation of Thakkar scattering factors in *NoSpherA2* and interface to *Olex2*

The new scattering factors were implemented in the *NoSpherA2* software (Kleemiss *et al.*, 2021 ▸; Kleemiss, 2019 ▸), which allows calculation and writing of atomic scattering factors to a .tsc file. A .tsc file contains individual atomic scattering factors of all atoms in a structure, referenced by their label. Kleemiss *et al.* (2021 ▸) previously defined the file format as a standard interface to *olex2.refine* for any type or origin of scattering factor. Through this interface, the newly proposed Thakkar scattering factor refinement can easily be performed from the *Olex2* graphical user interface (GUI) when *NoSpherA2* is enabled by selecting the ‘ThakkarIAM’ option as the source of a .tsc file. This direct integration means that the use of a .tsc file allows immediate switching between the classical Gaussian model and the ThakkarIAM models by checking or unchecking the use of *NoSpherA2* in the *Olex2* GUI after the .tsc file has been calculated once.

The calling of *NoSpherA2* to generate this file is usually handled by *Olex2*, but a manual operation of *NoSpherA2* to create scattering factors is also possible by calling *NoSpherA2* with the command line arguments -cif <*CIF-filename*> -xyz <*XYZ-filename*> -IAM -dmin <*resolution*> where a .cif file, an .xyz file and a resolution in floating point format must be specified instead of the placeholders. This program writes a .tsc file in the working directory, with all atoms in the .cif files addressed by labels and calculated scattering factors. Generating a .tsc file in this way takes less than a second, so in favour of tailor-made scattering factors for each scattering vector of the given structure, there is no permanently deposited table in *Olex2*. This approach allows for treating all atoms or individual parts of a crystal structure model, the latter when using the ‘hybrid’ mode (Jha *et al.*, 2023 ▸) within the GUI of *Olex2*. The hybrid mode allows the calculation of scattering factors for each part of a model by assigning parts with the PART command, as in *SHELXL* syntax, each of which is calculated independently, and then the resulting scattering factor files are automatically merged into a combined .tsc file. A combination of Hirshfeld atom refinement (HAR)-based results using *NoSpherA2* for a well defined molecule in the unit cell, in conjunction with the Thakkar scattering factors for a heavily disordered solvent molecule, is thus possible given the existing capability of merging .tsc files using *NoSpherA2*.

Access to the scattering factors of ions is given by appending ‘-Cations A B C’ and ‘-Anions A B C’ to the program call, where A B C can be a space-separated list, of any length, of atomic labels for which the corresponding ionic scattering factor is to be used. The neutral atomic wavefunction is used instead if this element has no ionic scattering factor.

*NoSpherA2* calls can be extended with the keyword ‘-ED’ to enable the calculation of electron diffraction scattering factors. When used within *Olex2*, the detection of electron diffraction data is automatically passed to the *NoSpherA2* calls, if applicable.

The value of the exponent of the radial wavefunction reported for hydrogen was manually changed during implementation. This adjustment was made to match better with the hydrogen atom scattering factor proposed by Stewart *et al.* (1965 ▸) by using an exponential parameter of 1.15 bohr instead of the analytical solution of 1.0 bohr. The resulting electron density matches that of hydrogen when bound to a second atom. This adjustment is consistent with the atom scattering factor of hydrogen used in *SHELXL* or *olex2.refine*, for example (Sheldrick, 2015 ▸; Bourhis *et al.*, 2015 ▸).

## 4. Software and refinements

All refinements throughout this work were performed with *Olex2* (Dolomanov *et al.*, 2009 ▸) using the refinement engine *olex2.refine* (Bourhis *et al.*, 2015 ▸). Where models with Slater-type densities were implemented, the user-supplied scattering factor interface within *NoSpherA2* was employed (Kleemiss *et al.*, 2021 ▸; Kleemiss, 2019 ▸). All maps and residual electron-density analyses were performed using tools within *Olex2*, including generating electron-density maps. The scattering factor plots were generated using *NoSpherA2* output files and *MatPlotLib* (Hunter, 2007 ▸).

To evaluate the performance of the new scattering factors obtained using Thakkar wavefunctions and the difference from the classical Gaussian fits, the results of refinements from four different structures analysed by X-ray diffraction are presented below, with increasing complexity of the structures: a small-molecule organic compound C_8_H_11_F_2_N_3_O, labelled **1** (Pattison *et al.*, 2009 ▸), a salt of an inorganic cation with an organic counterion and solvent water C_4_H_4_O_6_Ca·4H_2_O, labelled **2** (only present in *Olex2*), an early *d*-block element metal–organic complex C_16_H_16_CoF_6_N_4_O_4_S_2_, labelled **3** (Congreve *et al.*, 2003 ▸), and an osmium hexahydride bis-tri­phenyl­phosphane complex C_24_H_44_OsP_2_, labelled **4** (Kleemiss *et al.*, 2021 ▸). Data sets **1**–**3** are taken from the *Olex2* installation, as they are supplied with the software as examples. Data set **4** is the same as that used by Kleemiss *et al.* (2021 ▸). The chemical structural formulae of the refined compounds are shown in Scheme S1 in the supporting information.

All refinements for the 4G+c models were repeated from the deposited data sets using *olex2.refine* to ensure that any effects observed in this comparison were not due to differences in refinement software or truncated file precision when interfacing with fixed-format output programs. All hydrogen atoms were refined freely, removing any AFIX constraints from the refinements.

To test the implementation of the scattering factors derived for use with electron diffraction data, available data sets from the literature were used: nicotinic_acid_2x-merged from van Genderen *et al.* (2016 ▸), hereafter referred to as **5**, and CuPcCl16_ED_final from Gorelik *et al.* (2021 ▸), hereafter referred to as **6**. These two chemical structural formulae also are given in Scheme S1.

For **5**, the AFIX commands were changed to hydrogen distances according to averaged neutron diffraction data available in the literature, since it has already been shown that these cannot be refined at X-ray distances and, when freely refined, are located either at neutron distances or even further (Allen & Bruno, 2010 ▸; Klar *et al.*, 2023 ▸). The refinement of **5** using the scattering factors reported in the deposited files was carried out using *olex2.refine*, resulting in unusually high values of residual electrostatic potentials. Upon further investigation, the Gaussian scattering factors used in the deposited data were comparable neither in amplitude nor in radial behaviour to the scattering factors obtained by Peng (1999 ▸) or those derived in this work based on Thakkar wavefunctions. Comparison with the scattering factors of Maslen *et al.* (2016 ▸) revealed that X-ray scattering factors are present in the .cif and embedded .ins files, although the authors mention using Peng’s scattering factors in the article. Besides the mismatched scattering factors, the instruction file still contained coefficients for *f*′ and *f*′′ that are not even applicable to electron diffraction data. Therefore, a second model was refined by replacing the original scattering factors with those of Peng (1999 ▸), removing the anomalous dispersion parameters, and the results using these refinements are compared with those obtained using the putative X-ray scattering factors and the new Mott–Bethe transformed Thakkar scattering factors. All refinements of **5** were performed using values of *a* = 0.2 and *b* = 0.0 for the *SHELX*-type weighting scheme parameters during the refinement, as suggested by the *Olex2* routine, because optimizing the weighting scheme parameters would result in *a* > 0.2.

The refinement of **6** was performed using four fixed values of the *SHELX*-type weighting scheme coefficients *a* (*a* = 0.001, 0.1, 0.5, 1.0), while *b* was kept at 0. This approach was chosen after initial observations that the coefficients varied drastically between refinement iterations, and to emphasize the importance of the correct choice of weighting scheme coefficients in the kinematic refinement of ED data. All refinements were repeated with *olex2.refine*, using the Gaussian instructions deposited with the original data, to obtain comparable results regardless of the implementation of, for example, residual electron density and weighting scheme analysis.

## 5. Results and discussion

### 5.1. X-ray diffraction

A comparison of scattering factors obtained using Thakkar wavefunctions with those from the Gaussian fits (4G+c; Maslen *et al.*, 2006 ▸) is shown in Fig. 2 ▸ for six selected elements: H, C, O, P, Ca and Os in a range up to sin(θ)/λ = 2 Å ^−1^. Extended plots up to sin(θ)/λ = 4 Å ^−1^ are shown in Figs. S1 and S2. Additional plots of the respective singly charged anion and cation have also been included for elements with available wavefunctions. A complete list of available ions is given in Table S1. The difference between the neutral atom scattering factors for these elements is plotted in Fig. 3 ▸.

The overestimation of the scattering power of the 4G+c model at high scattering vectors (Fig. 3 ▸ and Fig. S2) is well known (Fox *et al.*, 1989 ▸), especially for heavy elements, where a different set of fitting functions based on a logarithmic scale is proposed for this region of the scattering vector magnitude. An extension using a fifth Gaussian function during the fit has been proposed by Waasmaier & Kirfel (1995 ▸), which can account much better for the behaviour at higher scattering vector magnitudes (compare Figs. S3 and S4). However, the differences at lower scattering vector magnitudes persist due to the different radial long-range behaviour of the Gaussian functions and the analytical Fourier transform of the Thakkar wavefunctions. Therefore, the differences shown in Fig. 2 ▸ at lower sin(θ)/λ values cannot be corrected by adding an extra function to the Gaussian fit.

Our refinements of the X-ray diffraction data for structures **1**–**4** demonstrate comparable performance between the two different spherical models in all refinements. The residual statistics differ by a maximum of 0.3‰ (see Table 1 ▸). A notable trend is the systematic improvement in *R* values and residual densities in all cases when using Thakkar-based scattering factors. As no additional parameters were introduced in the refinement, a comparison between the refinements can be made directly.

**Figure 4:**
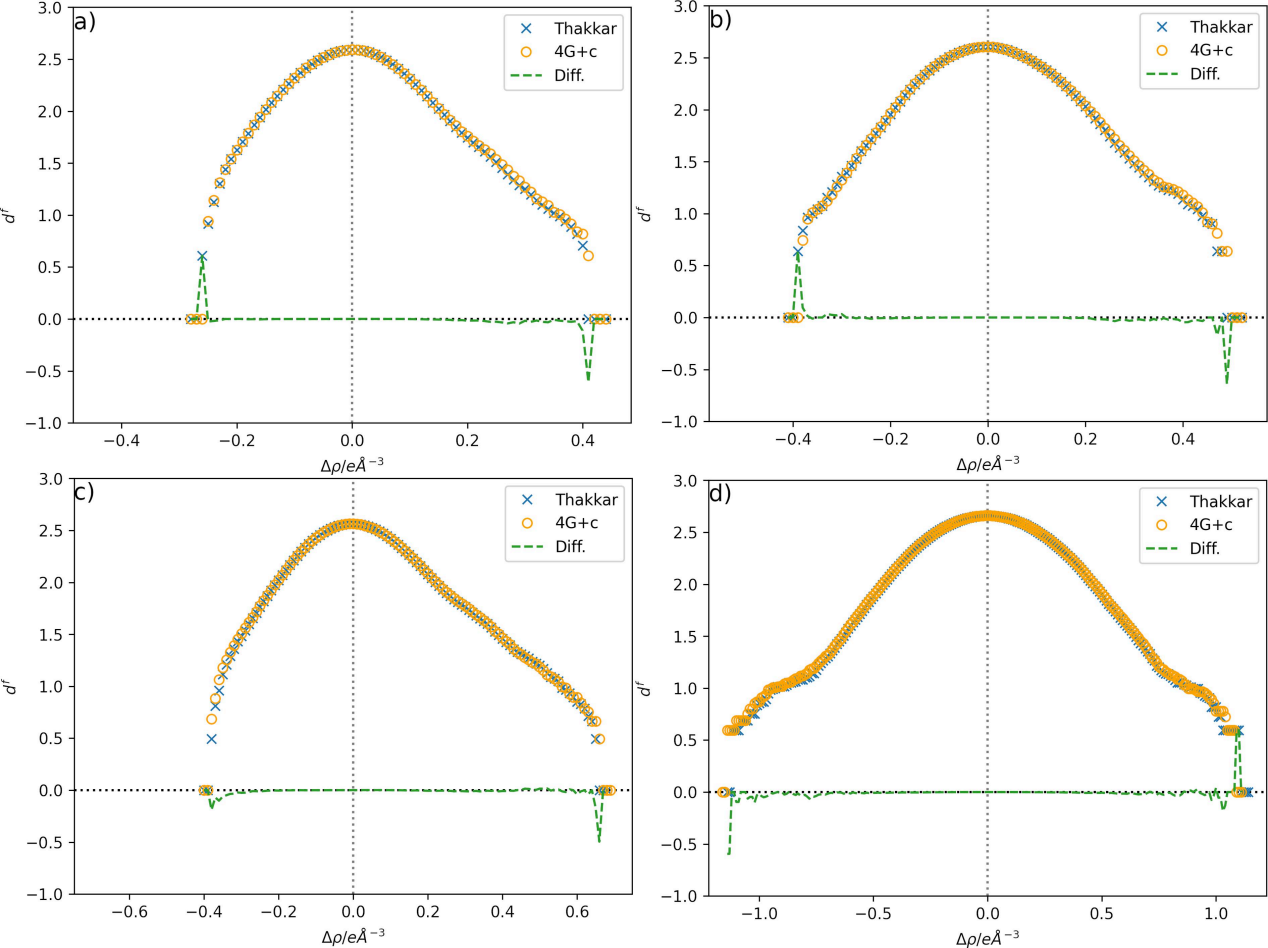
Fractal dimensional analysis (Meindl & Henn, 2008 ▸) of the residual densities of the models using 4G+c (orange circles; Maslen *et al.*, 2006 ▸) and the newly proposed Thakkar-wavefunction-based densities (blue crosses), and the difference between them (Thakkar − Maslen, green dashed line). (*a*) Structure **1** (*e*_gross_ = 44.6/44.4), (*b*) structure **2** (*e*_gross_ = 30.0/29.9), (*c*) structure **3** (*e*_gross_ = 37.9/37.7) and (*d*) structure **4** (*e*_gross_ = 176.6/175.6). *e*_gross_ is given in electrons and for 4G+c/Thakkar, respectively.

**Figure 5:**
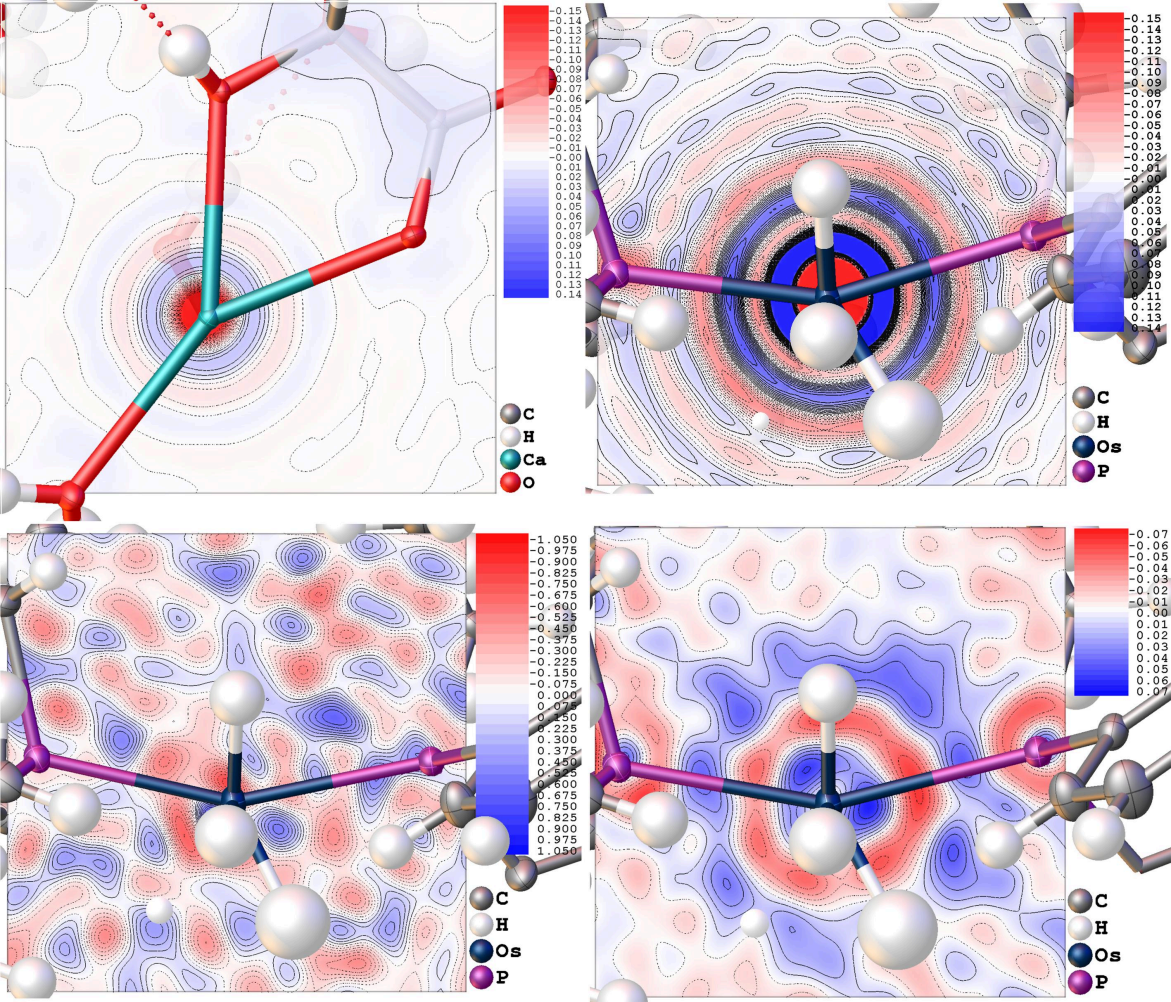
(Top row) Deformation electron density of (left) **2** in the O—Ca—O plane and (right) **4** in the P—Os—P plane in e Å^−3^, where the complex-valued *F*_c,Diff_ = *F*_c,Thakkar_ − *F*_c,4G+c_ was used for the Fourier synthesis of an electron-density map using identical positions and ADPs. Maps were calculated for the entire unit cell, and the contour planes and colour code are shown in e Å^−3^. The minimum, maximum and r.m.s. values of the deformation electron-density maps are: **2** −0.313, 0.037 and 0.006 e Å^−3^ and **4** −4.008, 0.382 and 0.037 e Å^−3^. (Bottom row, left) Residual electron density of **4** using Thakkar scattering factors (minimum, maximum and r.m.s.: −1.108, 1.125 and 0.175 e Å^−3^) and (right) the difference between residual densities (minimum, maximum and r.m.s.: −0.072, 0.074 and 0.011 e Å^−3^) using residual electron-density grids calculated for Thakkar and 4G+c after both refinements were individually converged and then subtracted from each other afterwards, thus taking into account the differences in both *U*_eq_ and the scattering factors between the two models.

A comparison between the model based on the Thakkar scattering factors and the 4G+c scattering factors using the average weighted root mean-square difference 〈wRMSD〉 of the structural parameters and their subsets (see Table 2 ▸) shows that the new scattering factors perform similarly for most light elements (*Z* < 35). Only the model for **4** shows a significant difference in the structural model after refinement: the atomic displacement parameters (ADPs) are significantly smaller [〈wRMSD(*U*_eq_)〉 is greater than 3] for the Os atom in the model using Thakkar-based scattering factors. The criterion for a significant difference is chosen when the 〈wRMSD〉 is greater than 1.41, which would correspond to the difference being more significant than the sum of both uncertainties, assuming that the uncertainties of both values are of comparable magnitude. *U*_eq_ of Os decreased from 0.01505 (2) to 0.01494 (2) Å^2^ when switching from the 4G+c model to the Slater-type densities. A complete list of atomic positions and ADPs of all structures is given in a spreadsheet deposited as supporting information. The change in the ADP of Os can be interpreted as a direct consequence of the difference in scattering power between the two models: the Thakkar-based scattering factor has a lower contribution over the whole scattering vector space (see Fig. 3 ▸). In the convoluted dynamic electron density, this effect is compensated for by the decrease in ADP, which gives a similar height to the electron-density peak at the atomic position. This ‘narrowed’ ADP will also cause other nearby atoms, such as the six bound H atoms, to shift. The H atoms in **4** bonded to carbon have a 〈wRMSD〉 of 0.370 in terms of their distance to the carbon atom and 0.0593 in terms of their value of *U*_iso_ in the two models, while the H atoms bound to Os have corresponding 〈wRMSD〉 values of 0.1905 and 0.3828, respectively, systematically showing smaller *U*_iso_ [0.051 (6) versus 0.054 (7) Å^2^] and longer distances for the model using Thakkar scattering factors [1.52 (2) versus 1.51 (2) Å]. The systematic difference in scattering factors observed in Fig. 3 ▸ can rationalize this small but systematic trend. During a least-squares refinement, the high-order reflections, for which the Thakkar scattering factors predict a lower scattering power, will lead to a reduction in the value of *U*_iso_, which will increase the contribution of this atom for high-order reflections. It might be expected that a different set of Slater-type wavefunctions might have different effects on the *U*_iso_ values depending on the near-atomic description of the electron density, especially concerning relativistic effects and the choice of basis sets, where a reduction in the extension of the radial function by a lower value of the exponent will increase the scattering factor for high-order reflections. A correlation between the changes in the electron-density description and the bond lengths of the hydrogen atoms is plausible, especially when comparing the radially shaped effects of residual electron density around the Os atom already discussed by Kleemiss *et al.* (2021 ▸) and the difference between the 4G+c model and the new Thakkar refinements visualized in Figs. 4 and 5. These residuals might overshadow the comparatively small scattering power of the hydrogen atom during the least-squares refinement, and therefore refine to values that position the hydrogen atom in one of the residual electron density maxima rather than its actual position.

To compare the models on the basis of the total distribution of residual electron-density differences in the unit cell and not just their minima and maxima, they were plotted on a grid and analysed according to the procedure described by Meindl & Henn (2008 ▸). The resulting plots are shown in Fig. 4 ▸. It is important to note that the residual electron-density analysis in Fig. 4 ▸ is performed on a more precise grid than the residual electron-density calculation used for peak search after refinements, which was used for the data reported in Table 1 ▸ (40 × 45 × 60 grid points are used after refinements for the residual electron-density peak search, and 120 × 144 × 180 for the fractal dimension analysis of *e.g.* **1**).

Noticeable differences can be observed at the highest absolute residual electron-density values, where a redistribution towards a more symmetrical distribution around the zero value can be seen. However, the differences are primarily subtle at the intermediate residual electron-density values compared with the most extreme ones. The most significant difference is found in **4**, where *e*_gross_ [as defined by Meindl & Henn (2008 ▸)] is reduced by 1.0 electron when using the Thakkar scattering factors compared with the 4G+c model. Otherwise, the two models perform similarly for spherical scattering factors, as expected from the similarity of the scattering factor values for the commonly available resolution of X-ray diffraction data shown in Fig. 2 ▸.

The concept of deformation densities can be applied to highlight further the difference between the two models and find where the most considerable difference in *e*_gross_ for **4** is located within the unit cell. In this case, the deformation is not between a non-spherical electron-density model and the corresponding spherical one, but between the calculated electron densities using Thakkar-based and 4G+c instruction-based scattering factors. The top row of Fig. 5 ▸ shows the difference in static electron density between the 4G+c and Thakkar-based structure factors on identical models, *i.e.* positions and ADPs. These densities are obtained with the fast Fourier transform map function of *cctbx* (Grosse-Kunstleve *et al.*, 2002 ▸), using the complex-valued result *F*_Diff_ = *F*_c,Thakkar_ − *F*_c,4G+c_ as structure factors. *F*_c,Thakkar_ and *F*_c,4G+c_ are complex-valued structure factors of the corresponding scattering factors. Using complex values for the structure factors of both models eliminates the necessity of assigning one model’s phases to a difference in moduli as would be the case for a residual density map. Regions where the Thakkar structure factors would give a higher electron density in an *F*_calc_ map are shown in blue, while the regions with reduced electron density are shown in red. A pattern similar to Fourier truncation ripples can be seen around the atom positions, most clearly around the heaviest element of each structure. This pattern results directly from the different Fourier behaviour of the two types of function used for the Fourier synthesis of the electron density. They lead to a pattern similar to the Gibbs phenomenon (Gibbs, 1898 ▸, 1899 ▸) due to the discontinuity of the constant term in the Gaussian model, in contrast to the Thakkar model which has no term giving rise to a Gibbs phenomenon. The magnitude of the scattering factors is similar for both models. The electron density obtained from the Thakkar scattering factors shows a systematic redistribution of the electron density, a behaviour that is well known from residual electron-density maps around heavy scatterers. The cusp versus delta distribution, and especially the slower long-range decay of the Slater electron density, lead to extreme difference values around the Ca and Os atoms (−0.313 e Å^−3^ for Ca and −4.008 e Å^−3^ for Os), assuming that identical atomic positions and displacement parameters are used during the Fourier synthesis.

The bottom row of Fig. 5 ▸ shows the residual electron density in the P—Os—P plane of **4** after refinement with the Thakkar scattering factors on the left, and the difference between the residual electron-density maps of the 4G+c model and the Thakkar model on the right, both in their respective converged model. The plot shows that, despite the differences in *U*_iso_ discussed above, a systematic pattern of differences remains. The differences in the scattering factors cannot be compensated by changing the ADPs since the displacement factor has a different radial behaviour from the scattering factors. The remaining difference is significantly lower in value than the residual electron density, but given the similar distance of the hydrogen atoms to the features of the residual difference maps, an effect on the refined values of the Os—H distances can be expected.

A table of direct comparison statistics for the models is available in the supporting information as Table S2.

The differences in both the absolute values of the scattering factors (Figs. 2 ▸ and 3 ▸) and the resulting model statistics (Tables 1 ▸ and 2 ▸) of these refinements appear to be small in comparison with the well established 4G+c formalism and might raise the question of whether the use of the Thakkar scattering factors is advantageous. However, the more evenly spaced residual electron-density maps (Figs. 4 ▸ and 5 ▸) and the more physically reasonable assumption of an electron-density distribution near the atomic position with a cusp provide a better description of the modelled electron density. For example, the average Os—H distance in the Thakkar refinements is 1.5206 ± 0.0225 Å, and that using the 4G+c fit is 1.5133 ± 0.0233 Å. On average, the Os—H distance was elongated by 7.3 mÅ using the Thakkar densities, which is indeed within the range of the standard uncertainty of around 23 mÅ, but a systematic trend in the refinements is observed.

### 5.2. Electron diffraction

**Figure 6:**
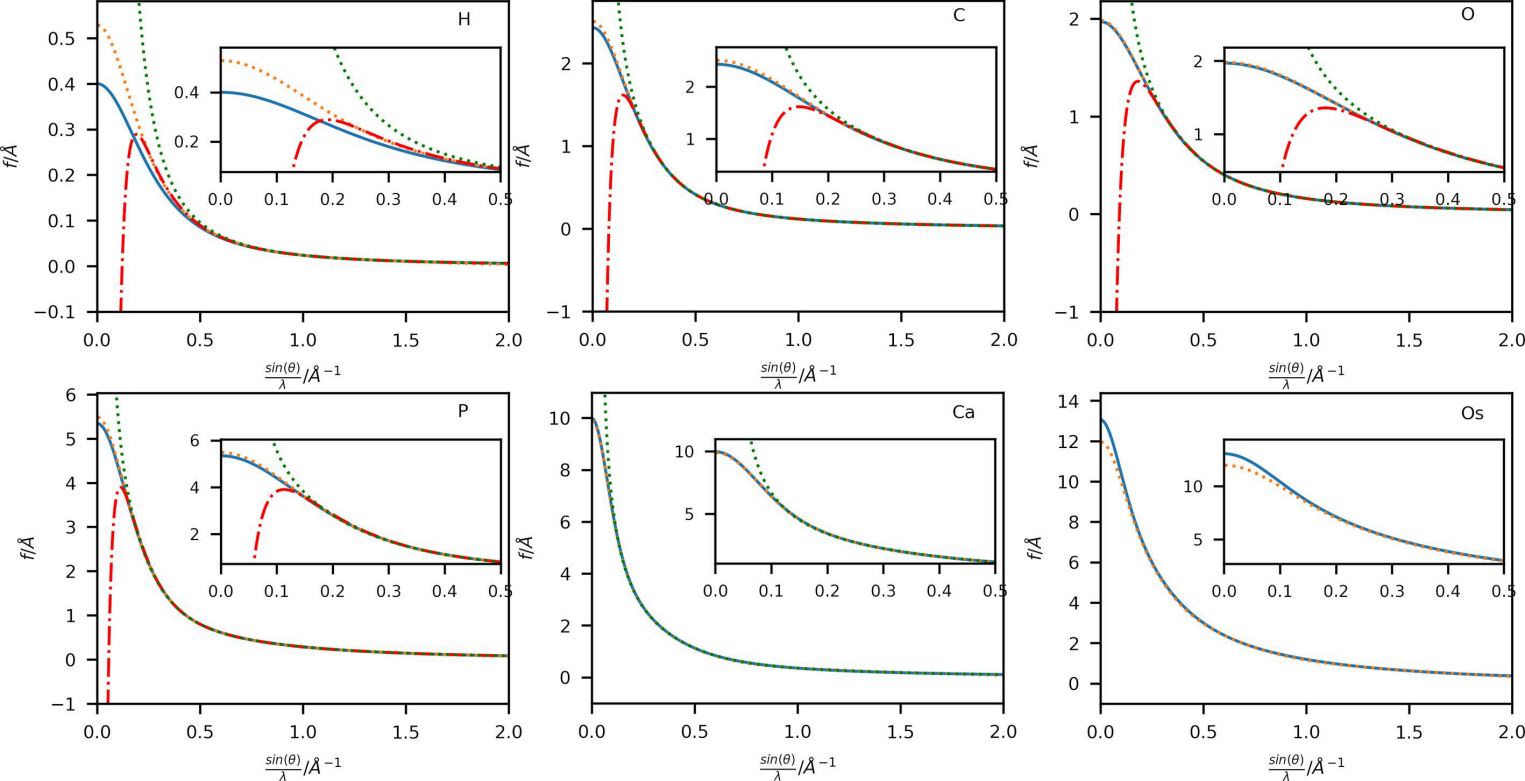
Plots of electron diffraction scattering factors (in ångströms) obtained by Peng (orange dotted lines; Peng, 1999 ▸) and Thakkar wavefunctions (this work; neutral: solid blue lines, cation: green dotted lines, anion: red dashed–dotted lines) for elements (top left to bottom right) H, C, O, P, Ca and Os against sin(θ)/λ in Å^−1^.

**Figure 7:**
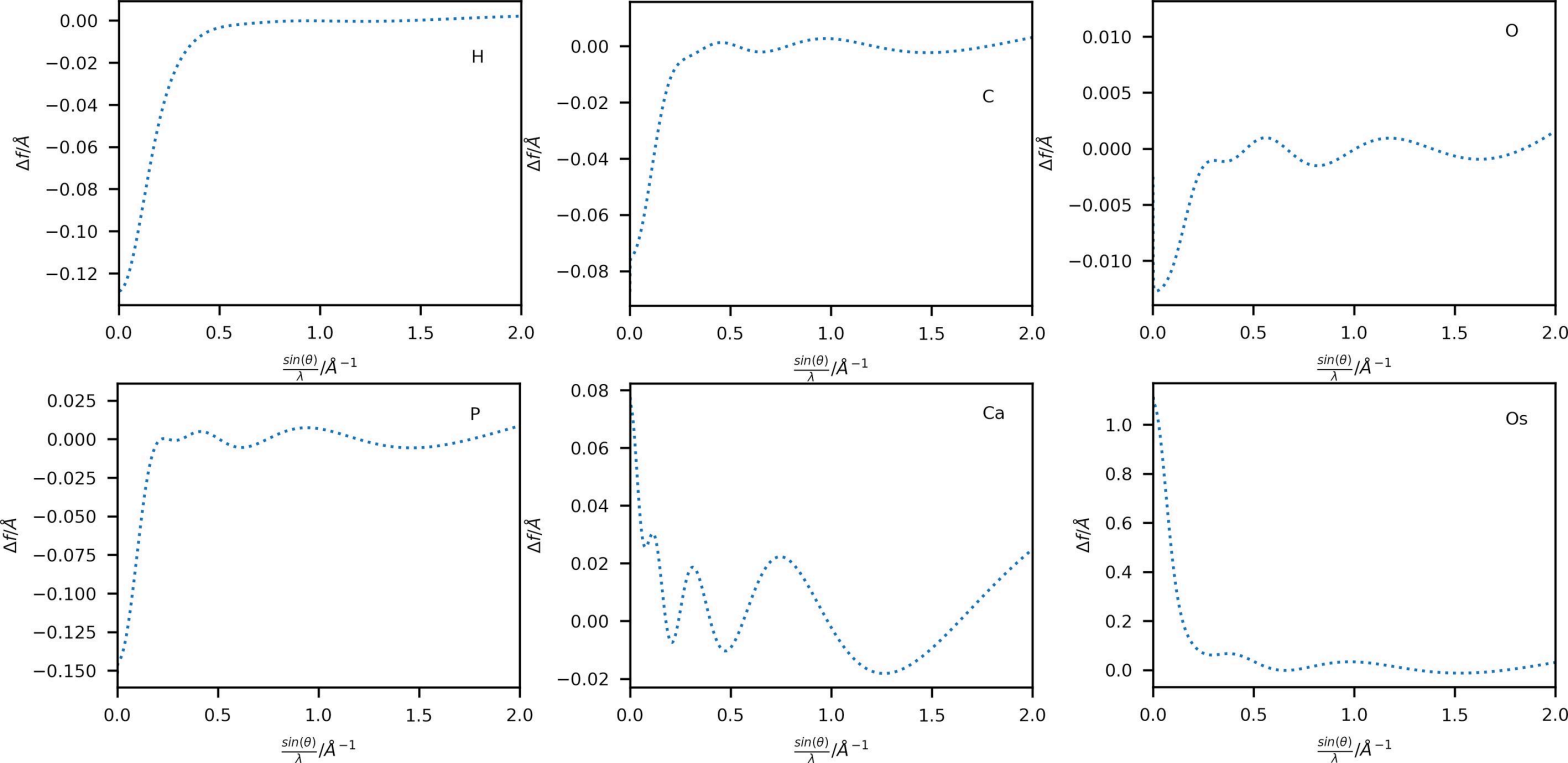
Plots of the difference between Thakkar-based electron scattering factors (this work) and those obtained by Peng (1999 ▸), both in ångströms, for neutral atoms of elements (top left to bottom right) H, C, O, P, Ca and Os against sin(θ)/λ in Å^−1^.

The scattering factors calculated from the wavefunctions by Koga *et al.* (1999 ▸, 2000 ▸) are analytically converted to electron diffraction scattering factors using equation (4). No interpolation or fitting is required since the transformation of equation (4) is exact and analytical. Where available, the resulting scattering factors of the neutral atoms and the ions are shown in Fig. 6 ▸, together with the scattering factors in the 4G formalism of Peng (1999 ▸). Their differences are plotted in Fig. 7 ▸. Extended versions of these plots up to sin(θ)/λ = 4.0 Å^−1^ are available in the supporting information (Figs. S5 and S6).

For the early elements of the periodic table up to P, there is a tendency for the scattering factor to be larger at low sin(θ)/λ values in Peng’s model. Hydrogen shows the most pronounced difference. However, this may be due to the choice of the radial exponent, according to Stewart *et al.* (1965 ▸), which gives a much narrower electron-density distribution. Following this discussion, the other elements showing similar overestimation by Peng (1999 ▸) at low sin(θ)/λ could be explained by the different long-range behaviour of Gaussian and Slater-type functions (Magalhães, 2014 ▸). Note the similarity of the scattering power of neighbouring elements such as C, N (not shown here) and O, and especially the inverse relation between the value of the scattering factors at sin(θ)/λ = 0 and the atomic number for these elements. X-ray crystallographers working with ED data may be misled into assigning a heavier atom when the opposite is true. *F*(000) is not directly proportional to the number of electrons of an element, as in the case of X-rays. However, drastic differences in scattering power are also observed for heavier elements such as Ca and Os, with relative differences up to 10% for the lowest sin(θ)/λ values in the case of Os.

The calculation of scattering factors for ions based on the available wavefunctions (see Table S1) yields very significant differences for the low-resolution region since the atom’s charge dominates the electrostatic potential at long distances. At very low resolutions, the type of atom is almost insignificant compared with the influence of charges (see Fig. 6 ▸). These drastic low-resolution effects highlight the importance of describing ED data regarding the (partial) charge of the atoms in their respective environment. At the high-resolution end of the calculated scattering factors, the differences between neutral and ionic species are minor, primarily due to the highly reduced scattering power of the atoms, even for heavy scatterers like Os, compared with their behaviour for X-rays. Peng shows that the scattering factors of ions can be modelled on the basis of the neutral electron scattering factor modified by a term due solely to the charge [equation (4) of Peng (1999 ▸)]. This model introduces an ‘effective’ nucleus charge in the Bethe transform. In Figs. S7 and S8 we show the scattering factors obtained with this effective nuclear charge and the differences obtained by this model calculation for C and O. The differences show a systematic overestimation of the coulombic term of the charged atom. This observation is understandable, since cations and anions rearrange their electronic structure upon ionization and do not have the same electrostatic potential near the nucleus as a neutral atom with a point charge added. Therefore, we do not recommend using scattering factors obtained with this model.

The scattering factors were used to refine the experimental electron diffraction data of structures **5** and **6** taken from the literature, as mentioned in Section 4. The data properties and the statistics of the refinement results are summarized in Table 3 ▸ for all the models built in this work.

**Figure 8:**
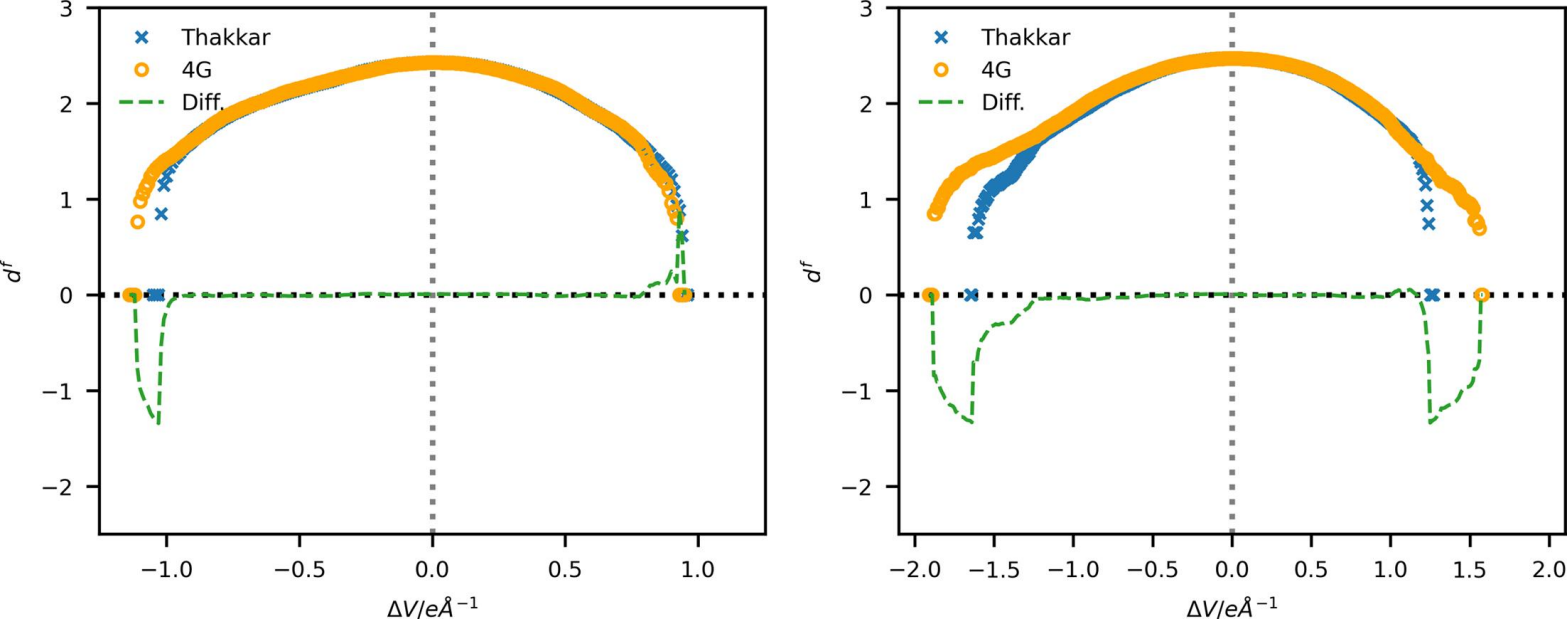
Fractal dimensional analysis of the residual electrostatic potential of the models using Gaussian instructions (orange circles) and the newly proposed Mott–Bethe transformed Thakkar wavefunction electrostatic potentials (blue crosses), and the difference between them (green dashed lines), (left) for **5**, and (right) for **6** with weighting parameter *a* = 1.0 and Peng (1999 ▸) scattering factors.

The refinement statistics of structure **5** with the two different tables appear surprisingly comparable in *R* statistics, but the residual electron-density scale in the case of the suspected X-ray scattering factor instructions reveals an apparent discrepancy in the model. This significant difference might be expected given the assumption of the X-ray scattering factors used. The analysis of the residual electrostatic potential maps of selected refinements from Table 3 ▸, according to the residual analysis of Meindl & Henn (2008 ▸), is shown in Fig. 8 ▸ and Fig. S10.

The refinement of structure **6** using the different values of the *SHELXL* weighting scheme parameter *a* shows how sensitive the refinement is to an improperly chosen value of the weighting scheme. In the *SHELXL*-type weighting scheme equation, setting the parameter *b* to 0 results in

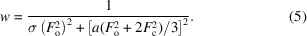




From equation (5), a plausible explanation for the observed effect on the refinements could be related to the magnitude of the 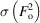 in relation to their measured intensity 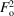 , staying within the kinematic model. If there is an underestimation of the value of 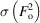 , the value of the resulting weight is significantly increased if there is no other contribution in determining weights, which is the case as the parameter *a* approaches zero. If there is a significant difference between the observed and calculated intensities, the term multiplied by *a* becomes relatively tiny compared with a case with matching values, effectively down-weighting the mismatched reflection. In a data set with incorrectly estimated measurement uncertainties, this can improve the refinement by giving more weight to reflections that match. However, it is easy to see that this could introduce a confirmation bias into the refinement. Therefore, a careful determination of 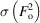 is imperative to ensure that the weighting scheme does not introduce a confirmation bias by artificially reducing the weight of disagreeing reflections. If 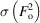 is poorly determined, the refinements will give these reflections where the uncertainty is underestimated an overestimated weight during the refinement and thus spoil the result. If the values of 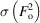 are correctly estimated, the effect of the weighting scheme should not be as dramatic as in this case. Therefore, **6** is assumed to have some reflections with poorly defined 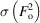 .

The refinement of **5** using the scattering factors of Peng was improved by switching to Slater-type scattering factors (compare Fig. 8 ▸, left). As in the X-ray examples, the residual distribution becomes more symmetric, reducing the occurrence of negative values in this case and adding some more positive regions. A statement about a measure similar to *e*_gross_ is not applicable since the electrostatic potential inside the unit cell is not strictly conserved as with electron density. In the refinement of **6**, a decrease in the residual electrostatic potential map is observed at both ends of the range. A similar improvement is also shown in the difference in *R* values in Table 3 ▸. The observed systematic improvement in the refinement statistics, in terms of both residual electrostatic potential and *R* values shown in Table 3 ▸, as the parameter *a* of the weighting scheme is increased over refinements of **6** is consistent with the assumption that the values of 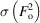 are underestimated. The increased weighting of reflections that are consistent with the structural model by the *SHELX*-type weighting scheme improves the refinement results by down-weighting disagreeing ones.

A refinement of **6** was also attempted using the scattering factors of charged Cu^+^, Cl^−^ and N^+^, but the refinement became unstable since the residuals of some *F*_c_ and *F*_o_ values became too large. In the model with neutral atom scattering factors, reflections with Miller indices 020, 040 and 110 have |*F*_c_|^2^ values of 724.44, 752.31 and 775.41, respectively. Using the same geometry and ADPs, a model with charged atomic scattering factors gives |*F*_c_|^2^ values of 969 242.0, 34 472.0 and 1 932 810.0, respectively. The extreme difference can be explained by considering the steep divergence of the scattering power at low sin(θ)/λ values for the charged atoms. Since the values of the scattering factors increase drastically, the current model leads to enormous values of the structure factor differences entering the least-squares matrix, which in this case becomes unstable and generates shifts in atomic positions and ADPs, making the next refinement cycle break.

The application of extreme values (*x* > 10 000) of the empirical extinction correction, as implemented in *SHELXL* or *olex2.refine*, reduces this instability. It brings the extreme *F*_c_ values for low resolution back to a similar scale to the *F*_o_ values, but for the wrong physical reason, and it should, therefore, be avoided. The empirical extinction formula in these programs multiplies *F*_c_ by λ^3^, which for the acceleration voltage used in **6** would be a factor of about 1.5 × 10^−5^ Å^3^. Therefore, it brings the total factor down to more reasonable values for *x* > 10 000. However, the extinction effect does not account for the increase in the measured intensity of weak reflections observed at the same time as the decrease in intensity of the most intense reflections. The effect of dynamic diffraction would have to be considered to redistribute the extremes of the strongest low-resolution reflections, but an application of this theory is beyond the scope of the current work.

In order to build stable models using the charged atoms in ED, it might be possible to use a damped refinement to adjust the model slowly to the sudden change in the scattering power of the introduced ions. Alternatively, a linear combination of interpolated scattering factors between the ions might be possible to introduce the charges gradually, rather than have an abrupt change in the model.

## 6. Conclusions

The newly implemented Thakkar-wavefunction-based scattering factors perform favourably when employed in refinements of X-ray diffraction data compared with scattering factors based on the 4G+c model. The more symmetric distribution of the residual densities (compare Table 1 ▸ and Fig. 4 ▸) suggests the suitability of the functionality for application in all structures, especially since scattering factors are available for all elements in the range *Z* = 1–103. The systematic change in the ADPs when using Slater-type densities, most pronounced in the heaviest elements, needs further investigation but could indicate a systematic overestimation of the ADPs using Gaussian models. The data quality of the presented refinements does not yet allow a significant judgement of the effect. More exact intensity data measurements will allow for a more significant judgement when the difference becomes more prominent than the refinement uncertainties. Here, highly redundant data with low background signal from photon-counting detectors will help reduce uncertainties in the refinement. This study did not use such data, in order to show the feasibility and performance of the new wavefunctions even with inferior or more routine data quality.

A different point that needs to be addressed in this context is the effect of the level of theory (including relativistic effects and electron correlation) and basis sets used in the Slater-type wavefunctions. The observed improvement upon using Thakkar wavefunctions can be attributed to the relationship between the constant of the Gaussian fits, which, upon Fourier transformation, becomes a delta function located at the core, and the more accurate cusp description in the Slater-type wavefunctions. The delta function behaves very differently from the analytical transform of the Slater-type wavefunctions. While the difference in electron density between the Gaussian and Slater models does not explain all the differences in the experimental data, it still shows the systematic patterns common to compounds containing heavy elements. The remaining effects could partially be explained by a different radial behaviour of a dispersion correction term used in crystallographic models, a second constant within the framework of structural model building, which is the subject of ongoing investigations.

Given this observation, the use of Thakkar densities for the Hirshfeld partitioning within *NoSpherA2*–HAR, where spherical pro-molecule densities are calculated using the Thakkar densities (Kleemiss *et al.*, 2021 ▸), is justified. Furthermore, it raises the question of whether HAR could perform even better if the quantum mechanical calculations were performed using Slater-type basis sets. Given the popularity and success of Gaussian functions in quantum mechanical software packages, implementing a Slater-type package in *NoSpherA2* is only a question of availability and open access to the software. Possible software in this regard is *ADF* (Software for Chemistry & Materials BV, Amsterdam, The Netherlands), commercial software unavailable to the authors.

The availability of the newly presented scattering factors within the framework of *Olex2*/*NoSpherA2* allows the provision of analytically derived scattering factors for all purposes, even in combination with non-spherical scattering factors, due to the .tsc file. This file format also allows usage outside *Olex2*. The availability of ions, where available in the atomic wavefunctions of Koga *et al.* (1999 ▸, 2000 ▸) (compare Table S1), greatly enhances the applicability to ionic structures, especially in electron diffraction. If a combination of these scattering factors with dynamic diffraction theory can be applied, the large values of *F*_c_ observed in these structures would, within the framework of the Bloch-wave formalism, lead to a strongly affected structure matrix, which would lead to an enhancement of the dynamic effect and influence parameters such as the refined thickness. Including charged scattering factors would most likely allow for much better refinement results, since most atoms in chemical species carry a charge simply due to the difference in electronegativity, even in highly covalent bonds. However, it should be noted that integer charges of atoms are an artificial model, and a model employing partial charges based on the valence situation, like the case in HAR or transferable aspherical atom model techniques, would be even more applicable. The question about the partitioning scheme used during quantum-mechanics-based refinements like HAR could be addressed using high-quality ED data when dynamic effects and other currently approximated effects are included in the model.

The reported improvements in residual electrostatic potential within this work using the kinematic model of electron diffraction are to be considered only a step in the right direction. They should not be seen as a reason to avoid using dynamic diffraction models. Only a combination of correct scattering factors with appropriate diffraction theory will yield applicable models for refining electron diffraction data completely.

## 7. Related literature

For further literature related to the supporting information, see Baddour (2010 ▸), Michels (2021 ▸) and Watson (1952 ▸).

## Supplementary Material

Zip archive containing CIFs of different models for structures 1-4. DOI: 10.1107/S1600576723010981/nb5360sup1.zip

Spreadsheet with complete structural results and ESDs after refinement with both models. DOI: 10.1107/S1600576723010981/nb5360sup2.xlsx

Additional figures, mathematical derivation and tables. DOI: 10.1107/S1600576723010981/nb5360sup3.pdf

## Figures and Tables

**Table 1:** Data properties and refinement statistics of X-ray data refinements using Thakkar scattering factors and 4G+c for structures **1** to **4**

Structure	**1**	**2**	**3**	**4**
〈*I*/σ〉	17.8	18.2	27.1	40.3
*d*_min_	0.7	0.62	0.77	0.58
No. of reflections (all, unique, unique *I* > 2σ)	25 545, 5668, 4491	9682, 4066, 3768	11 963, 4897, 4121	199 465, 13 109, 11 278
No. of parameters	255	179	310	315
Space group	*Pna*2_1_	*P*2_1_2_1_2_1_	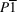	*P*2_1_/*n*

Refinement results: Thakkar (this work)				
*R*_1_ (*I* > 2σ)	0.0384	0.0317	0.0309	0.0211
*wR*_2_(all)	0.0959	0.0663	0.0820	0.0367
Maximum and minimum residuals (e Å^−3^)	0.360, −0.245	0.413, −0.365	0.537, −0.357	0.898, −0.964

Refinement results: 4G+c				
*R*_1_ (*I* > 2σ)	0.0387	0.0320	0.0311	0.0211
*wR*_2_(all)	0.0967	0.0666	0.0826	0.0369
Maximum and minimum residuals (e Å^−3^)	0.364, −0.242	0.422, −0.377	0.548, −0.372	0.881, −0.972

**Table 2:** Averaged wRMSD between models employing Thakkar and 4G+c scattering factors, models *a* and *b* 〈wRMSD〉 (unitless) is calculated as 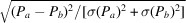 running over all parameters *P*, using fractional coordinates for position parameters; *U*_*ii*_ and *U*_*ij*_ once without (*i* ≠ *j*) and once with diagonal values of anisotropically refined atoms; and *U*_eq_ of anisotropic atoms or *U*_iso_ of isotropic atoms of all atoms (including hydrogen atoms) in the asymmetric unit. The second values after the slash are for the heaviest scatterers present in the structure; if multiples of the same element are found, arithmetic averaging has been performed (4 F atoms in **1**, Ca in **2**, Co in **3** and Os in **4**). Here, *U*_eq_ refers to the mean value of *U*_*ii*_ in the Cartesian setting, as indicated by _atom_site_U_iso_or_equiv in .cif files.

	〈wRMSD〉, all atoms / heaviest scatterer(s)			
Structure	**1**	**2**	**3**	**4**
Position	0.0829 / 0.0357	0.0690 / 0.0234	0.0512 / 0.0268	0.1581 / 0.0556
*U_ii_*	0.1262 / 0.3216	0.0879 / 0.8739	0.1100 / 0.8761	0.4152 / 3.7051
*U_ij_* (*i* ≠ *j*)	0.0411 / 0.0237	0.0222 / 0.0237	0.0384 / 0.1627	0.0710 / 0.3101
*U_ij_*	0.0836 / 0.1726	0.0551 / 0.4488	0.0742 / 0.5194	0.2431 / 2.0076
*U*_eq_ or *U*_iso_	0.0833 / 0.3196	0.0757 / 0.8688	0.0572 / 0.7045	0.2322 / 3.8983

**Table 3:** Data properties and refinement statistics of ED data sets **5** and **6** The original authors reported Gaussian models of **5** with scattering factors (van Genderen *et al.*, 2016 ▸; Maslen *et al.*, 2006 ▸), and instructions were provided according to Peng (1999 ▸). Note how the low values of the weighting scheme parameter show the breakdown of the intended purpose in this case, as *wR*_2_ is much lower than *R*_1_.

Structure	**5**		**6**			
〈*I*/σ〉	4.8		4.7			
*d*_min_	0.75		0.81			
No. of reflections (all, unique, unique *I* > 2σ)	1353, 552, 303		4986, 1332, 1114			
No. of parameters	39		133			
Space group	*P*2_1_/*c*		*C*2/*m*			
*a* (weighting)	0.2		0.001	0.1	0.5	1.0

Thakkar (this work)						
*R*_1_ (*I* > 2σ)	0.3153		0.2928	0.2862	0.2645	0.2621
*wR*_2_(all)	0.6539		0.1232	0.6478	0.6757	0.6846
Maximum and minimum residuals (e Å^−1^)	0.935, −1.014		2.228, −1.771	1.626, −2.153	1.226, −1.898	1.245, −1.631

Gaussian models						
Scattering factors used	Maslen *et al.* (2006 ▸)	Peng (1999 ▸)	Peng (1999 ▸)			
*R*_1_ (*I* > 2σ)	0.3416	0.3195	0.3966	0.3066	0.2653	0.2665
*wR*_2_(all)	0.6923	0.6579	0.1481	0.6734	0.6748	0.6882
Maximum and minimum residuals (e Å^−1^)	3.004, −3.211	0.913, −1.062	6.357, −2.574	1.987, −1.981	1.681, −1.696	1.547, −1.723

## References

[bb1] Allen, F. H. & Bruno, I. J. (2010). *Acta Cryst.* B**66**, 380–386. 10.1107/S0108768110012048 20484809

[bb2] Avery, J. & Watson, K. J. (1977). *Acta Cryst.* A**33**, 679–680.

[bb3] Baddour, N. (2010). *J. Opt. Soc. Am. A*, **27**, 2144–2155. 10.1364/JOSAA.27.002144 20922005

[bb4] Bethe, H. (1930). *Annalen Phys.* **397**, 325–400.

[bb5] Bourhis, L. J., Dolomanov, O. V., Gildea, R. J., Howard, J. A. K. & Puschmann, H. (2015). *Acta Cryst.* A**71**, 59–75. 10.1107/S2053273314022207 PMC4283469 25537389

[bb6] Clementi, E. & Roetti, C. (1974). *At. Data Nucl. Data Tables*, **14**, 177–478.

[bb7] Congreve, A., Kataky, R., Knell, M., Parker, D., Puschmann, H., Senanayake, K. & Wylie, L. (2003). *New J. Chem.* **27**, 98–106.

[bb8] Coppens, P., Guru Row, T. N., Leung, P., Stevens, E. D., Becker, P. J. & Yang, Y. W. (1979). *Acta Cryst.* A**35**, 63–72.

[bb9] Cromer, D. T. & Mann, J. B. (1967). *X-ray Scattering Factors Computed from Numerical Hartree–Fock Wavefunctions.* Los Alamos Scientific Laboratory Report LA-3816. Los Alamos National Laboratory, New Mexico, USA.

[bb10] Cromer, D. T. & Mann, J. B. (1968). *Acta Cryst.* A**24**, 321–324.

[bb11] Cromer, D. T. & Waber, J. T. (1965). *Acta Cryst.* **18**, 104–109.

[bb12] Dolomanov, O. V., Bourhis, L. J., Gildea, R. J., Howard, J. A. K. & Puschmann, H. (2009). *J. Appl. Cryst.* **42**, 339–341.

[bb13] Fox, A. G., O’Keefe, M. A. & Tabbernor, M. A. (1989). *Acta Cryst.* A**45**, 786–793.

[bb47] Genderen, E. van, Clabbers, M. T. B., Das, P. P., Stewart, A., Nederlof, I., Barentsen, K. C., Portillo, Q., Pannu, N. S., Nicolopoulos, S., Gruene, T. & Abrahams, J. P. (2016). *Acta Cryst.* A**72**, 236–242. 10.1107/S2053273315022500 PMC4770873 26919375

[bb14] Gibbs, J. W. (1898). *Nature*, **59**, 200.

[bb15] Gibbs, J. W. (1899). *Nature*, **59**, 606.

[bb16] Gorelik, T. E., Habermehl, S., Shubin, A. A., Gruene, T., Yoshida, K., Oleynikov, P., Kaiser, U. & Schmidt, M. U. (2021). *Acta Cryst.* B**77**, 662–675.

[bb17] Grosse-Kunstleve, R. W., Sauter, N. K., Moriarty, N. W. & Adams, P. D. (2002). *J. Appl. Cryst.* **35**, 126–136.

[bb18] Guillot, B., Viry, L., Guillot, R., Lecomte, C. & Jelsch, C. (2001). *J. Appl. Cryst.* **34**, 214–223.

[bb19] Hansen, N. K. & Coppens, P. (1978). *Acta Cryst.* A**34**, 909–921.

[bb20] Hunter, J. D. (2007). *Comput. Sci. Eng.* **9**, 90–95.

[bb21] Jha, K. K., Kleemiss, F., Chodkiewicz, M. L. & Dominiak, P. M. (2023). *J. Appl. Cryst.* **56**, 116–127. 10.1107/S1600576722010883 PMC9901929 36777135

[bb22] Klar, P. B., Krysiak, Y., Xu, H., Steciuk, G., Cho, J., Zou, X. & Palatinus, L. (2023). *Nat. Chem.* **15**, 848–855. 10.1038/s41557-023-01186-1 PMC10239730 37081207

[bb23] Kleemiss, F. (2019). *NoSpherA2*, https://github.com/FlorianKleemiss/NoSpherA2.

[bb24] Kleemiss, F., Dolomanov, O. V., Bodensteiner, M., Peyerimhoff, N., Midgley, L., Bourhis, L. J., Genoni, A., Malaspina, L. A., Jayatilaka, D., Spencer, J. L., White, F., Grundkötter-Stock, B., Steinhauer, S., Lentz, D., Puschmann, H. & Grabowsky, S. (2021). *Chem. Sci.* **12**, 1675–1692. 10.1039/d0sc05526c PMC8179328 34163928

[bb25] Koga, T., Kanayama, K., Watanabe, S. & Thakkar, A. J. (1999). *Int. J. Quant. Chem.* **71**, 491–497.

[bb26] Koga, T., Kanayama, K., Watanabe, T., Imai, T. & Thakkar, A. J. (2000). *Theor. Chim Acta*, **104**, 411–413.

[bb27] Lentzen, M. (2019). *Acta Cryst.* A**75**, 861–865. 10.1107/S2053273319012191 PMC6833978 31692461

[bb28] Macchi, P. & Coppens, P. (2001). *Acta Cryst.* A**57**, 656–662. 10.1107/s0108767301010182 11679695

[bb29] Magalhães, A. L. (2014). *J. Chem. Educ.* **91**, 2124–2127.

[bb30] Malaspina, L. A., Wieduwilt, E. K., Bergmann, J., Kleemiss, F., Meyer, B., Ruiz-López, M. F., Pal, R., Hupf, E., Beckmann, J., Piltz, R. O., Edwards, A. J., Grabowsky, S. & Genoni, A. (2019). *J. Phys. Chem. Lett.* **10**, 6973–6982. 10.1021/acs.jpclett.9b02646 31633355

[bb31] Mann, J. B. (1967). *Atomic Structure Calculations. I. Hartree–Fock Energy Results for the Elements Hydrogen to Lawrencium.* Los Alamos Scientific Laboratory Report LA-3690. Los Alamos National Laboratory, New Mexico, USA.

[bb32] Mann, J. B. (1968). *Atomic Structure Calculations. II. Hartree–Fock Wavefunctions and Radial Expectation Values: Hydrogen to Lawrencium*. Los Alamos Scientific Laboratory Report LA-3691. Los Alamos National Laboratory, New Mexico, USA.

[bb33] Maslen, E. N., Fox, A. G. & O’Keefe, M. A. (2006). *International Tables for Crystallography*, Vol. C, pp. 554–590 Chester: Inter­national Union of Crystallography.

[bb34] McLean, A. D. & McLean, R. S. (1981). *At. Data Nucl. Data Tables*, **26**, 197–381.

[bb35] Meindl, K. & Henn, J. (2008). *Acta Cryst.* A**64**, 404–418. 10.1107/S0108767308006879 18421130

[bb36] Michels, A. (2021). *Magnetic Small-Angle Neutron Scattering: A Probe for Mesoscale Magnetism Analysis*. Oxford Academic.

[bb37] Mott, N. F. (1930). *Proc. R. Soc. London Ser. A*, **127**, 658–665.

[bb38] Olukayode, S., Froese Fischer, C. & Volkov, A. (2023*a*). *Acta Cryst.* A**79**, 59–79. 10.1107/S2053273322010944 36601764

[bb39] Olukayode, S., Froese Fischer, C. & Volkov, A. (2023*b*). *Acta Cryst.* A**79**, 229–245. 10.1107/S205327332300116X 36999622

[bb40] Pattison, G., Sandford, G., Yufit, D. S., Howard, J. A. K., Christopher, J. A. & Miller, D. D. (2009). *J. Org. Chem.* **74**, 5533–5540. 10.1021/jo9006943 19518071

[bb41] Pawlędzio, S., Malinska, M., Kleemiss, F., Grabowsky, S. & Woźniak, K. (2022). *IUCrJ*, **9**, 497–507. 10.1107/S2052252522005309 PMC9252150 35844484

[bb42] Pawlędzio, S., Malinska, M., Woińska, M., Wojciechowski, J., Andrade Malaspina, L., Kleemiss, F., Grabowsky, S. & Woźniak, K. (2021). *IUCrJ*, **8**, 608–620. 10.1107/S2052252521004541 PMC8256711 34258009

[bb43] Peng, L.-M. (1999). *Micron*, **30**, 625–648.

[bb44] Sheldrick, G. M. (2015). *Acta Cryst.* C**71**, 3–8.

[bb45] Stewart, R. F., Davidson, E. R. & Simpson, W. T. (1965). *J. Chem. Phys.* **42**, 3175–3187.

[bb46] Thorkildsen, G. (2023). *Acta Cryst.* A**79**, 318–330. 10.1107/S2053273323003996 PMC10317139 37265051

[bb48] Volkov, A., Macchi, P., Farrugia, L. J., Gatti, C., Mallinson, M. A., Richter, T. & Kritsanszky, T. (2016). *XD2016*, https://www.chem.gla.ac.uk/~louis/xd-home/.

[bb49] Waasmaier, D. & Kirfel, A. (1995). *Acta Cryst.* A**51**, 416–431.

[bb50] Watson, G. N. (1952). *A Treatise on the Theory of Bessel Functions*, 2nd ed. Cambridge University Press.

[bb51] Yonekura, K., Matsuoka, R., Yamashita, Y., Yamane, T., Ikeguchi, M., Kidera, A. & Maki-Yonekura, S. (2018). *IUCrJ*, **5**, 348–353. 10.1107/S2052252518005237 PMC5929380 29755750

