# Supplementary material for: Refinement of X-ray and electron diffraction crystal structures using analytical Fourier transforms of Slater-type atomic wavefunctions in Olex2
Source: J Appl Crystallogr. 2024 Feb 1;57(Pt 1):161–74. doi: 10.1107/S1600576723010981 (PMC10840308; doi:10.1107/S1600576723010981)
Supplement: Supplementary file 3 [file j-57-00161-sup3.pdf]

JOURNAL OF APPLIED CRYSTALLOGRAPHY

Volume 57 (2024)

Supporting information for article:

Refinement of X-ray and electron diffraction crystal structures using analytical Fourier transforms of Slater-type atomic wavefunctions in *Olex2*

Florian Kleemiss, Norbert Peyerimhoff and Michael Bodensteiner

Supplementary material for "**Refinement of X-ray and Electron diffraction crystal structures using analytical Fourier transforms of Slater-type atomic wavefunctions in Olex2**"

F. Kleemiss[a,b,*], N. Peyerimhoff[c], M. Bodensteiner[b]
florian.kleemiss@ac.rwth-aachen.de

[a]RWTH Aachen University, Institut für Anorganische Chemie, Landoltweg 1a, 52074 Aachen, Germany
[b]Universität Regensburg, Universitätsstraße 31, 93053 Regensburg, Germany
[c]University of Durham, Stockton Road, Durham DH1 3LE, United Kingdom

Derivation of Fourier Transform for radial functions

This presentation follows the argumentation of (Baddour, 2010; Michels, 2021). For a sufficiently decaying function $f: \mathbb{R}^3 \to \mathbb{C}$, we define its Fourier transform $F: \mathbb{R}^3 \to \mathbb{C}$ as (in agreement with Int. Tables Vol. B, 1.3.2.4.1)

$$F(\boldsymbol{\xi}) = \int_{\mathbb{R}^3} f(z) \exp\big(-2\pi i(\boldsymbol{\xi} \cdot z)\big)\, dz. \tag{S1}$$

Assume that f is a real-valued radial function that is: $f(z) = f_0(|z|)$ with a suitable function $f_0 : [0, \infty) \to \mathbb{R}$. In this case, the Fourier transform F of f takes the form

$$F(\boldsymbol{\xi}) = \int_0^\infty f_0(r) \left(\int_0^{2\pi} \int_0^\pi \exp\Big(-2\pi i\big(\boldsymbol{\xi} \cdot z(r, \alpha, \beta)\big)\Big) \sin\alpha\, d\alpha\, d\beta\right) r^2\, dr, \tag{S2}$$

with

$$z(r, \alpha, \beta) = r(\cos\beta \sin\alpha, \sin\beta \sin\alpha, \cos\alpha). \tag{S3}$$

Moreover, since the volume element dz is invariant under orthogonal transformations, the Fourier transform F of such a radial function f is again radial and depends only on $\xi = |\boldsymbol{\xi}|$, and we have

$$F(\boldsymbol{\xi}) = F\big((0,0,\xi)\big) = \int_0^\infty f_0(r) \left(2\pi \int_0^\pi \exp(-2\pi i \xi r \cos\alpha) \sin\alpha\, d\alpha\right) r^2\, dr. \tag{S4}$$

Since the Bessel function J_ν for $\nu > -\frac{1}{2}$ is given by the following relation (see Watson, 1952, p.47)

$$J_\nu(x) = \frac{\left(\frac{x}{2}\right)^\nu}{\Gamma\left(\nu + \frac{1}{2}\right)\Gamma\left(\frac{1}{2}\right)} \int_0^\pi \cos(x \cos\alpha) \sin^{2\nu}\alpha\, d\alpha, \tag{S5}$$

and since $J_{\frac{1}{2}}(x) = \sqrt{\frac{2}{\pi x}} \sin x$ (Watson, 1952, p.54), we end up with the relation

$$\int_0^\pi \exp(-2\pi i \xi r \cos\alpha) \sin\alpha\, d\alpha = \int_0^\pi \cos(2\pi\xi r \cos\alpha) \sin\alpha\, d\alpha = \sqrt{\frac{1}{\xi r}} J_{\frac{1}{2}}(2\pi\xi r) = \frac{\sin(2\pi\xi r)}{\pi\xi r}. \tag{S6}$$

These relations imply that the Fourier transform of a radial function $f: \mathbb{R}^3 \to \mathbb{R}$ with corresponding $f_0: [0, \infty) \to \mathbb{R}$ can be written as follows in terms of a Hankel transform of $\sqrt{r} f_0(r)$ of order ½:

$$F(\boldsymbol{\xi}) = \frac{2\pi}{\sqrt{\xi}} \int_0^\infty f_0(r) J_{\frac{1}{2}}(2\pi\xi r) r^{\frac{3}{2}} dr = \frac{2}{\xi} \int_0^\infty f_0(r) \sin(2\pi\xi r)\, r dr \tag{S7}$$

with $\xi = |\boldsymbol{\xi}| \neq 0$. In the case $\xi = 0$ we have

$$F(0,0,0) = 4\pi \int_0^\infty f_0(r) r^2 dr. \tag{S8}$$

Exercising this for a (hypothetical) atom with o=1, N=1, c=1, j_{max}=1 following definitions of symbols in equation (1) in the main article, choosing the radial electron density $\rho(r)$ as the function $f_0(r)$ yields:

$$f_0(r) \coloneqq \rho(r) = \frac{1}{4\pi} r^{2n-2} \exp(-2zr) \tag{S9}$$

$$F(\boldsymbol{\xi}) = \frac{1}{2\pi\xi} \int_0^\infty r^{2n-1} \exp(-2zr) \sin(2\pi\xi r)\, dr \tag{S10}$$

The resulting term $F(\boldsymbol{\xi})$ corresponds to the atomic scattering factor $f(k)$ in the main article, when choosing $\boldsymbol{k} = 2\pi\boldsymbol{\xi}$ and usually in crystallography $\xi = \frac{\sin(\theta)}{\lambda}$.

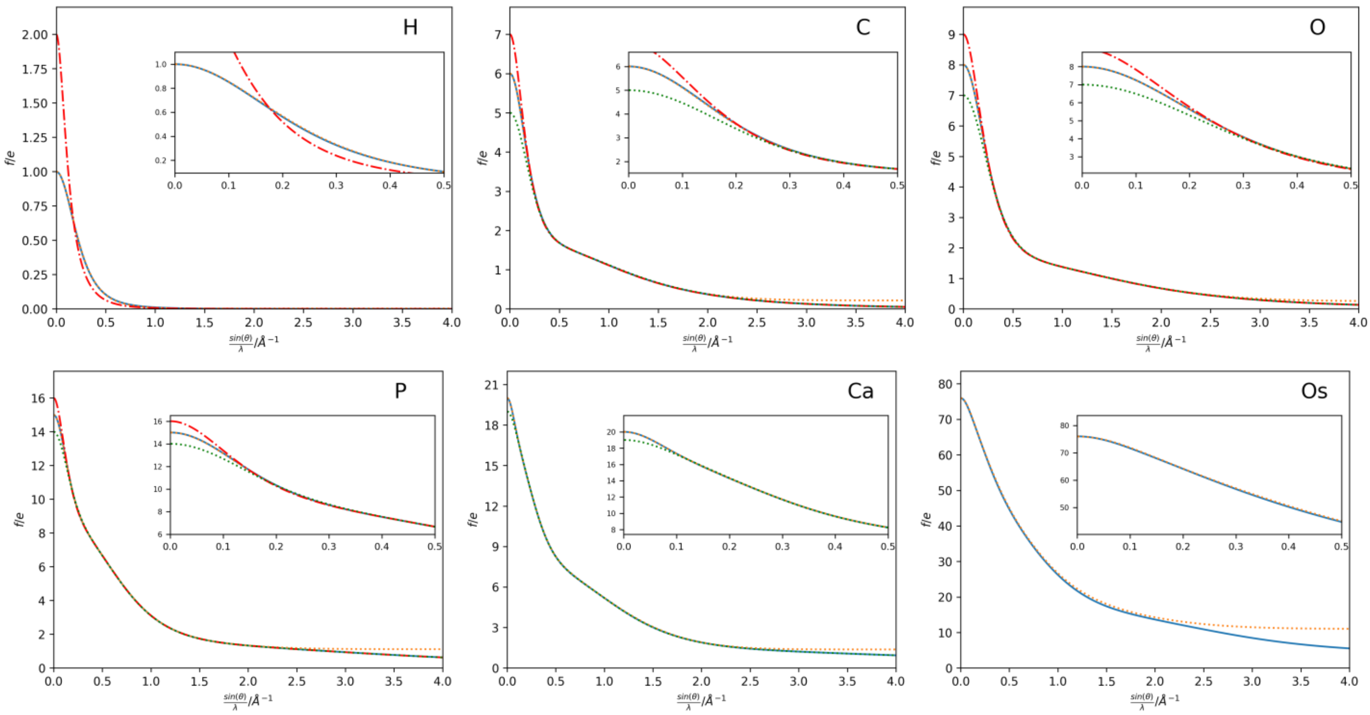

Figure S1: Plots of X-ray scattering factors in electrons obtained by 4G+c (orange, dashed lines; Maslen et al., 2006) and Thakkar wavefunctions (neutral: blue, solid, cation: green, dotted, anion: red, dashed-dotted) for elements (top left to bottom right): H, C, O, P, Ca, Os against an extended range of sin(θ)/λ in Å $^{-1}$.

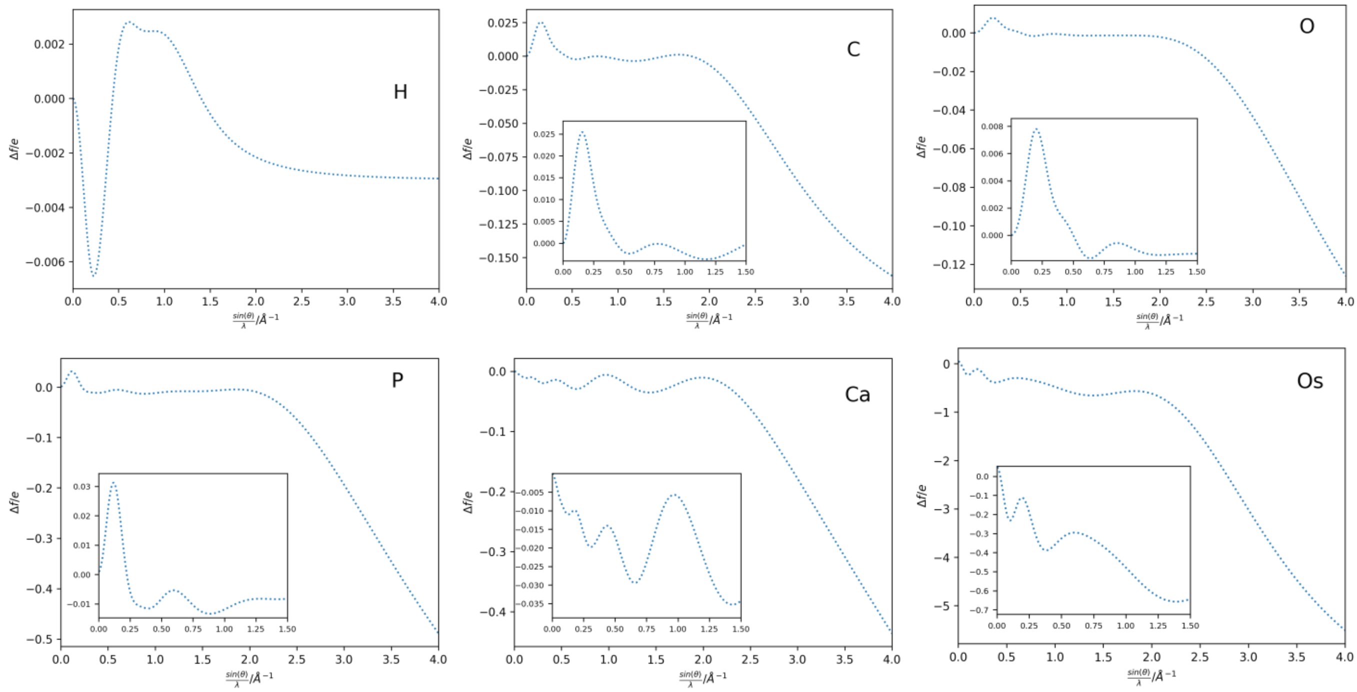

Figure S2: Difference plots between Thakkar-based X-ray scattering factors in electrons and those obtained by 4G+c (Maslen et al., 2006) for neutral atoms of elements (top left to bottom right): H, C, O, P, Ca, Os against an extended range of sin(θ)/λ in Å $^{-1}$.

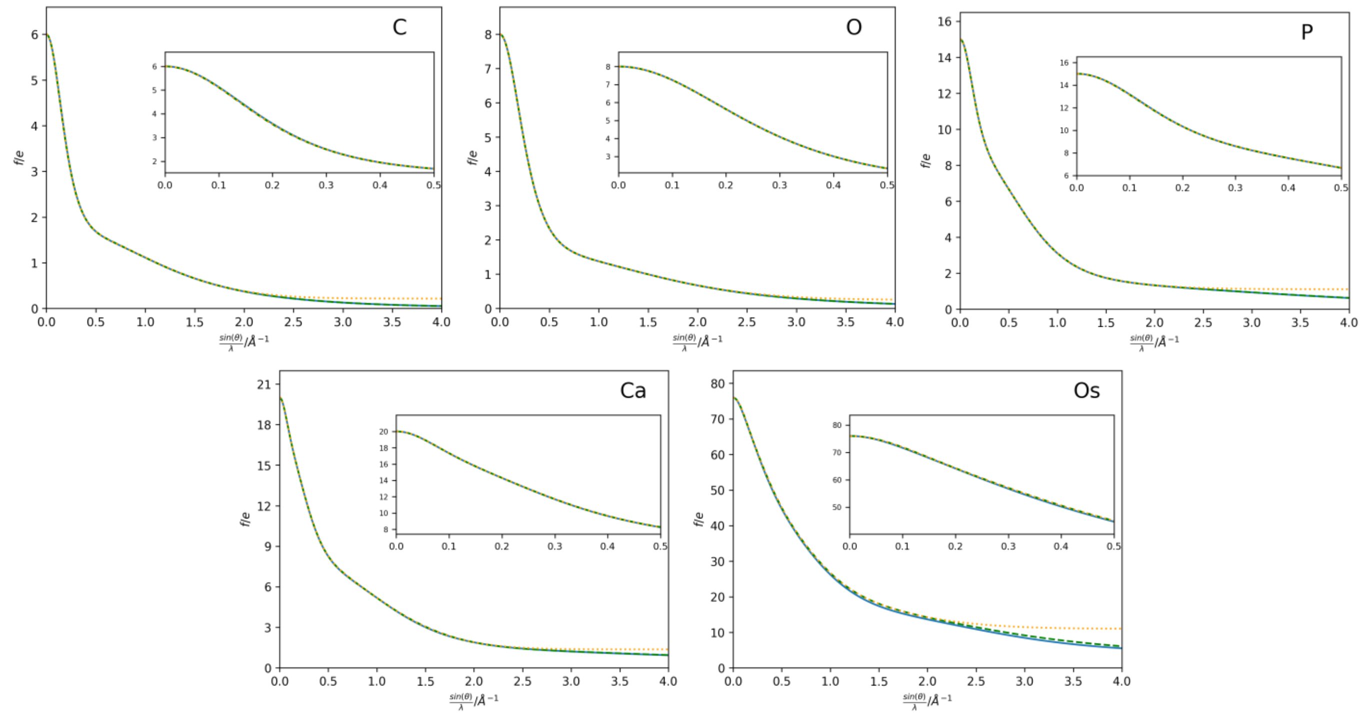

Figure S3: Plots of X-ray scattering factors in electrons obtained by 4G+c (orange, dotted lines; Maslen *et al.*, 2006), 5G+c fit (green, dashed; Waasmaier & Kirfel, 1995) and Thakkar wavefunctions (this work, neutral: blue, solid) for elements (top left to bottom right): C, O, P, Ca, Os against sin(θ)/λ in Å $^{-1}$. The scattering factor for H was not reported with 5 Gaussians in Waasmaier & Kirfel (1995).

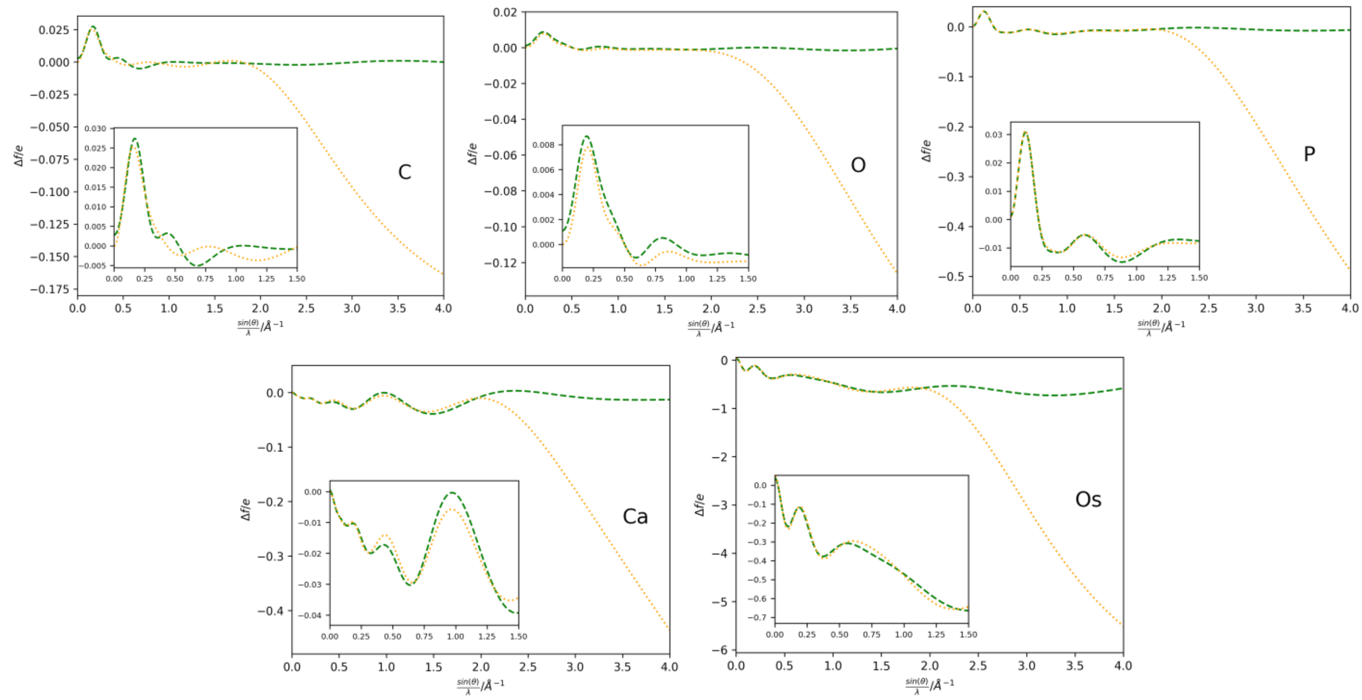

Figure S4: Plots of the difference between Thakkar-based X-ray scattering factors (this work) and those obtained by the 4-G+c fit (orange, dotted; Maslen *et al.*, 2006) and 5G+c fit (green, dashed; Waasmaier & Kirfel, 1995) in electrons for neutral atoms of elements (top left to bottom right): C, O, P, Ca, Os against sin(θ)/λ vector in Å $^{-1}$.

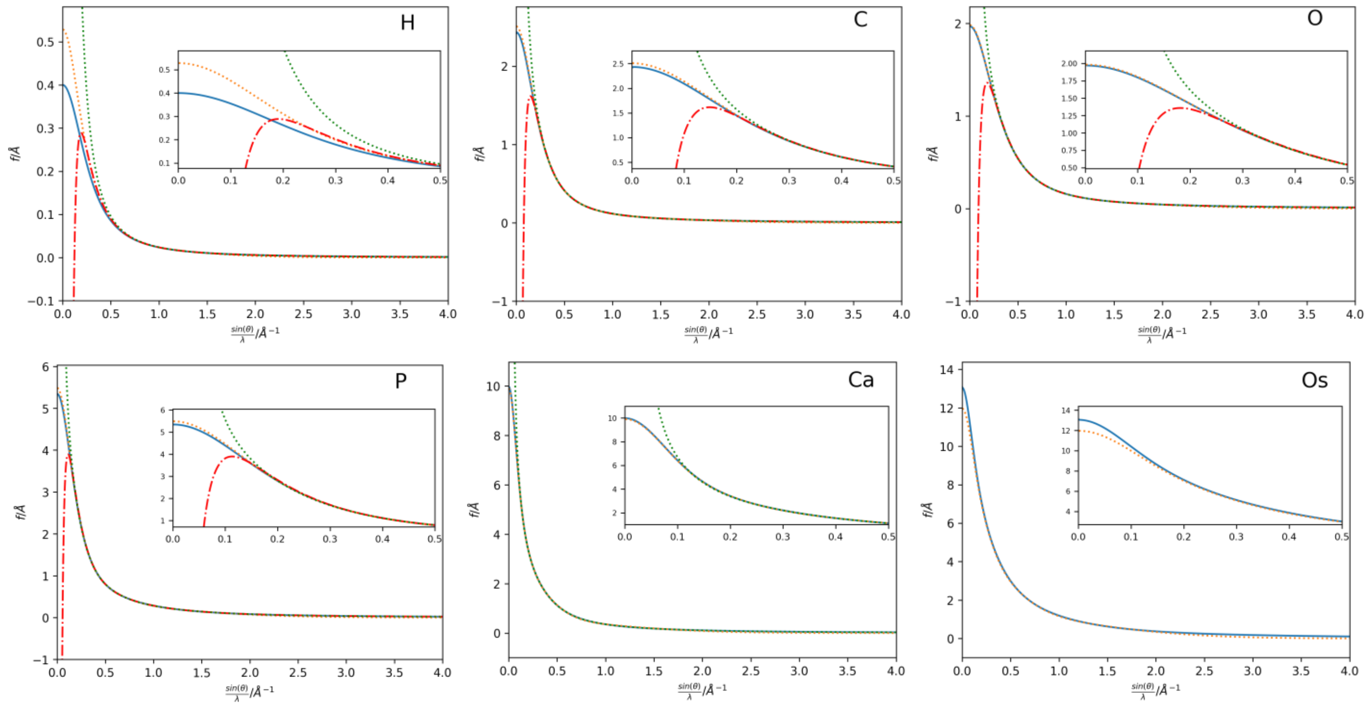

Figure S5: Plots of electron diffraction scattering factors in Å obtained by Peng (orange dotted; Peng, 1999) and Thakkar wavefunctions (this work, neutral: blue solid, cation: green dotted, anion: red dash-dot) for elements (top left to bottom right): H, C, O, P, Ca, Os against sin(θ)/λ in $Å^{-1}$.

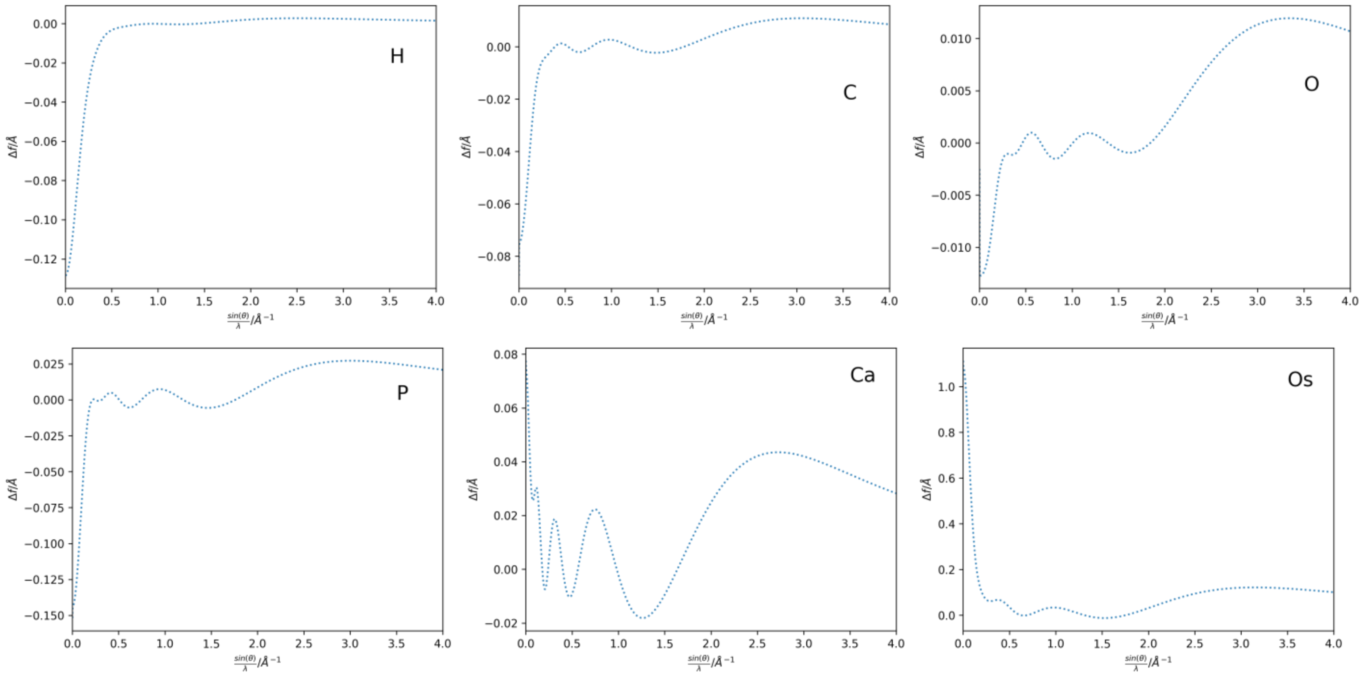

Figure S6: Plots of the difference between Thakkar-based electron scattering factors (this work) and those obtained by Peng (Peng, 1999) in Å for neutral atoms of elements (top left to bottom right): H, C, O, P, Ca, Os against sin(θ)/λ in $Å^{-1}$.

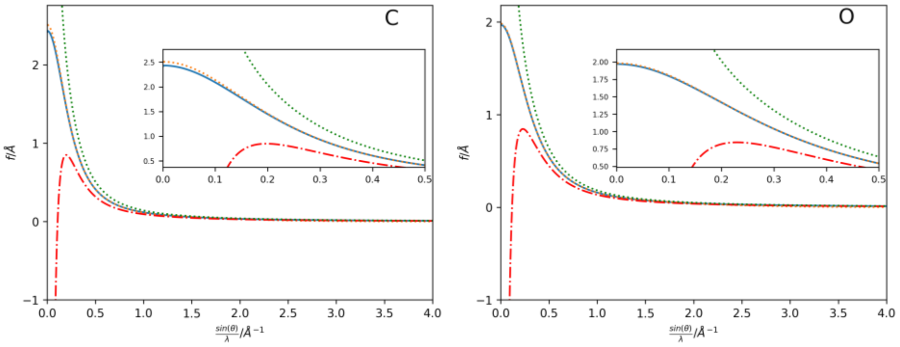

Figure S7: Plots of electron diffraction scattering factors in Å obtained by Peng (orange, dashed lines; Peng, 1999) and Thakkar wavefunctions (this work, neutral: blue, solid, cation-model: green, dotted, anion-model: red, dash-dot) for elements C (left) and O (right).

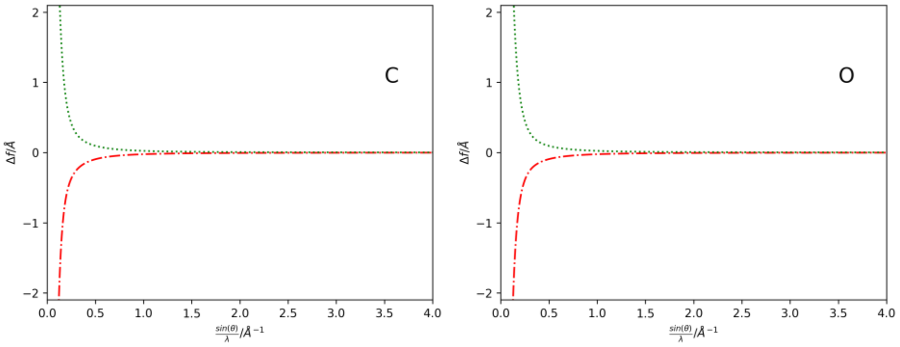

Figure S8: Plots of the difference between Thakkar-based X-ray scattering factors of wavefunctions calculated for Ions (see Figure 5) and the model using neutral atoms and an effective nuclear charge (Peng, 1998; see Figure S3) for C (left) and O(right).

Table S1: Table of Elements and the availability of their wavefunctions for the singly charged ions by Koga & Thakkar (Koga et al., 1999, 2000). An "x" marks a present and implemented entry.

Element	Cation	Anion	Element	Cation	Anion	Element	Cation	Anion
H		x	Ca	x		Sr	x	
Li	x	x	Sc	x	x	Y	x	x
Be	x		Ti	x	x	Zr	x	x
B	x	x	V	x	x	Nb	x	x
C	x	x	Cr	x	x	Mo	x	x
N	x	x	Mn	x	x	Tc	x	x
O	x	x	Fe	x	x	Ru	x	x
F	x	x	Co	x	x	Rh	x	x
Ne	x		Ni	x	x	Pd	x	x
Na	x	x	Cu	x	x	Ag	x	x
Mg	x		Zn	x		Cd	x	
Al	x	x	Ga	x	x	In	x	x
Si	x	x	Ge	x	x	Sn	x	x
P	x	x	As	x	x	Sb	x	x
S	x	x	Se	x	x	Te	x	x
Cl	x	x	Br	x	x	I	x	x
Ar	x		Kr	x		Xe	x	
K	x	x	Rb	x	x	Cs	x	

Table S2: Table of R1 between $F_{Thakkar}$ and $F_{4G(+c)}$, min, max and RMS values of deformation densities $\Delta\rho = (\rho_{Thakkar} - \rho_{4G(+c)})$, as those in Figure 5 of the main manuscript for all structures presented in the main text between the Gaussian description and the newly proposed Thakkar scattering factors. Identical models were used to calculate maps and structure factors; the difference originates only from the scattering factors. Unit of Min/Max/RMS is $eÅ^{-3}$ for **1** - **4** and Å for **5** and **6**.

Measure	1	2	3	4	5 (X-ray, Maslen et al., 2006)	5 (Peng 1999)	6 (a=1.0)
R1 (%)	0.169	0.205	0.236	0.744	73.194	1.652	0.379
Max	0.049	0.037	0.050	0.384	5.751	0.037	0.047
Min	-0.025	-0.313	-0.196	-4.113	-12.410	-0.083	-0.040
RMS	0.005	0.006	0.005	0.037	1.412	0.016	0.013

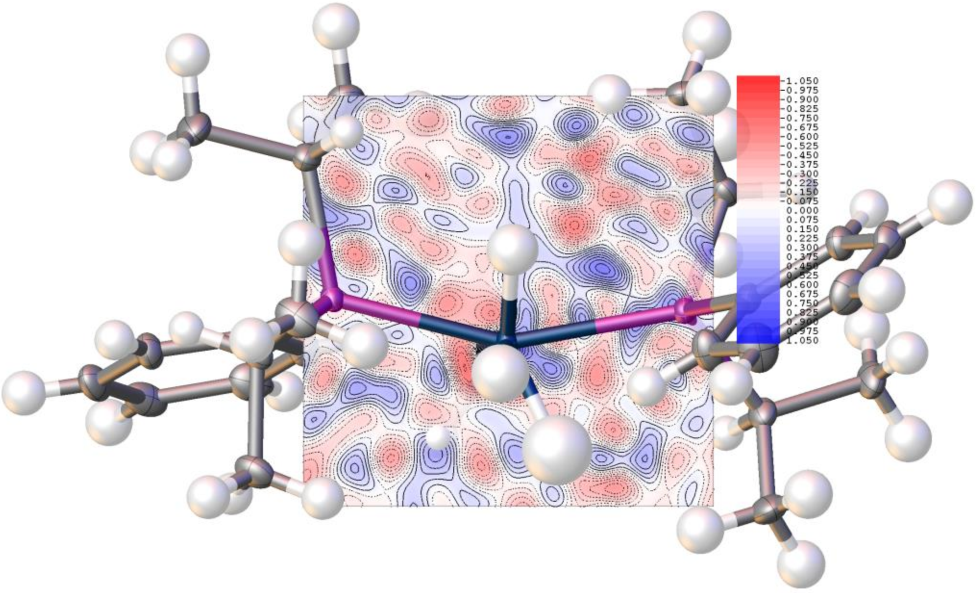

Figure S9: Residual electron density of **4** using 4G+c scattering factors (Min/Max/RMS: -1.192/1.399/0.176);

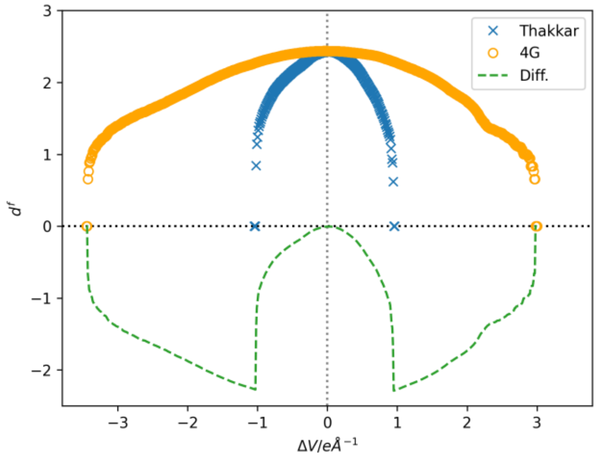

Figure S10: Fractal dimensional analysis of the residual electrostatic potential of the models using Maslen et al. 2016 X-ray Gaussian instructions (orange spheres) and the newly proposed Mott-Bethe transformed Thakkar wavefunction electrostatic potentials (blue crosses) and the difference between them (green dashed line) for **5**.

The extreme difference in the residual electrostatic potential distribution in the refinement using the scattering factors of Maslen et al. (Figure S10) is to be expected, given that they were intended for a different type of radiation. Surprisingly, the refinement was stable, and the molecular structure is still recognizable.

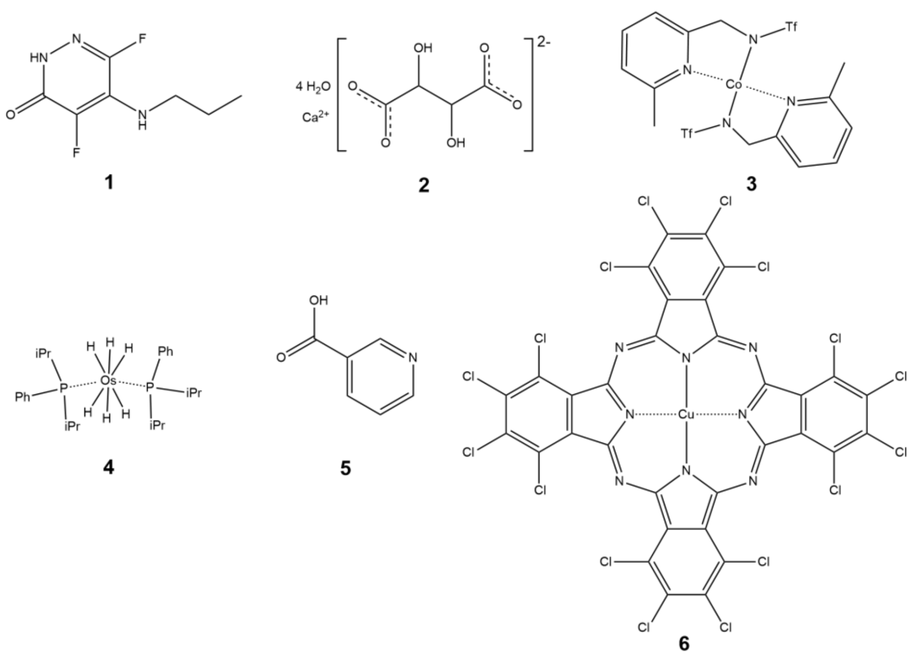

Scheme S1: Chemical structural formulae of the compounds **1** – **6** used during this work. The numbers below refer to the label used throughout the main text.

References

Baddour, N. (2010). *J. Opt. Soc. Am. A.*, **27**, 2144-2155.

Michels, A. (2021). *Magnetic Small-Angle Neutron Scattering: A Probe for Mesoscale Magnetism Analysis*, Oxford Academic, Oxford.

Watson, G. N. (1952) *A Treatise on the Theory of Bessel Functions, 2nd Edition*, Cambridge University Press, Cambridge.
